# Materials designed to degrade: structure, properties, processing, and performance relationships in polyhydroxyalkanoate biopolymers

**DOI:** 10.1039/d4py00623b

**Published:** 2024-10-15

**Authors:** Jessica N. Lalonde, Ghanshyam Pilania, Babetta L. Marrone

**Affiliations:** a Department of Mechanical Engineering and Materials Science, Duke University Durham NC 27708 USA; b Bioscience Division, Los Alamos National Laboratory Los Alamos NM 87545 USA blm@lanl.gov; c GE Aerospace Research Niskayuna NY 12309 USA

## Abstract

Conventional plastics pose significant environmental and health risks across their life cycle, driving intense interest in sustainable alternatives. Among these, polyhydroxyalkanoates (PHAs) stand out for their biocompatibility, degradation characteristics, and diverse applications. Yet, challenges like production cost, scalability, and limited chemical variety hinder their widespread adoption, impacting material selection and design. This review examines PHA research through the lens of the classical materials tetrahedron, exploring property-structure-processing-performance (PSPP) relationships. By analyzing recent literature and addressing current limitations, we gain valuable insights into PHA development. Despite challenges, we remain optimistic about the role of PHAs in transitioning towards a circular plastic economy, emphasizing the need for further research to unlock their full potential.

## Abstract 2

A materials tetrahedron framework is applied to PHA biopolymers to highlight design challenges and frame potential future research exploring structure, property, processing, and performance relationships.

## Graphical abstract

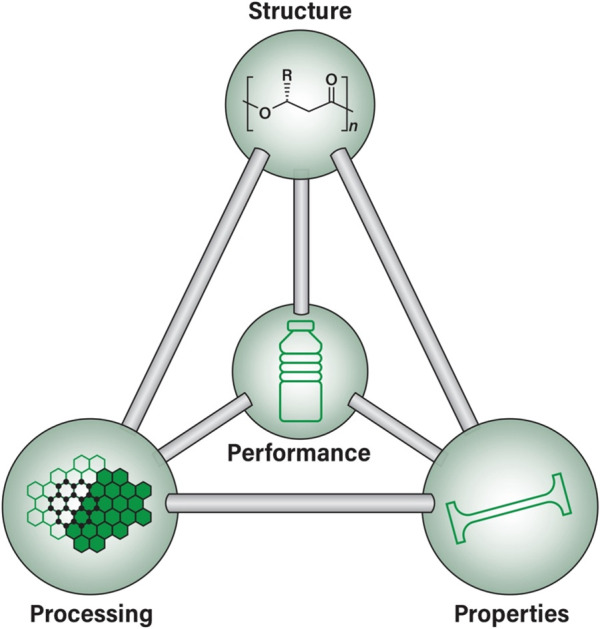

## 1 Introduction

Plastics derived from fossil fuels have become essential to society and modern daily life. However, the detriments to both environmental and human health throughout their entire material life cycle have devastating impacts.^1–3^ While the continued use of petroleum to synthesize and process single-use plastic is itself harmful, the end life-stage of plastic is also of paramount concern. As conventional plastics degrade in the environment, their decomposition has been known to produce microplastics.^4^ These materials have already affected global soils, which cycle further into the water, air, and atmosphere, and into our food chain.^4^ Conventional plastics such as low density polyethylene (LDPE), polypropylene (PP), polyvinyl chloride (PVC), and polystyrene (PS), are non-biodegradable and have been shown to remain in the environment in various forms for over 450 years.^5,6^

In addition, single-use petrochemical plastic use has continued to increase, particularly in recent years during the COVID-19 pandemic by reliance on plastic disposable items for safety and hygienic purposes.^7^ In addition, it has been reported that over 20 million metric tons of plastic waste, or 11% of the total plastic waste generated, directly entered aquatic ecosystems around the globe in 2016, with that number predicted to nearly double by 2030.^6^ Nearly 400 million metric tons of plastic are annually produced worldwide as of 2023 from raw petrochemical feedstocks.^2,8^ The management of this waste generated from raw feedstock has led to compounded economic, environmental, and health safety challenges.^8–10^ For instance, only a small fraction of the total amount of virgin plastic, ranging from 5–30%, is available for re-purposing or recycled for further use. This in turn generates a loss of up to 95% of the original economic value for the material.^5,9,11–14^

This has led to much economic opportunities lost for a potential circular “bio-plastic” economy. Even with increasing global efforts to manage plastic waste pollution, ongoing studies of the complex social, economic, and scientific issues of plastic waste management indicate that predicted growth in plastic waste generation exceeds current efforts for mitigating overall plastic pollution.^6,15^ These results indicate that urgent transformative changes in both materials design and policies are needed to help address this crisis.^2,8,14,16^

These ongoing concerns have generated strong interest in biologically sourced and biodegradable polymers as alternatives to conventional plastics. One model for potential future materials use includes the ultimate goal of creating a more circular, sustainable plastic economy in which the idea of waste is re-imagined.^4^ In this model of bio-based plastic substitutes, the waste products of end-of-life biopolymers can ultimately be re-used as raw feedstocks, as opposed to a more linear model, such as with conventional plastic, in which the material is simply unusable with current recycling infrastructure and technology or otherwise discarded.^17–21^

Polyhydroxyalkanotes (PHAs) are one example of a family of biologically produced polyesters that have been extensively investigated for their benefits as plastic alternatives.^22–26^ These well-known biopolymer materials, first developed in the early 20th century, are synthesized and accumulate in microbes in the presence of excess carbon sources and nutrients, acting as an energy storage mechanism.^27,28^ PHAs have often been selected and utilized as a model biologically-sourced polyester for their attractive and versatile qualities. Some of these qualities include robust biocompatibility, non-toxic degradation products, and chemical tunability. In addition, PHAs also have a unique opportunity to be recycled or reused by both chemical and biological means at the end of their life cycle for further use.^1,23,26,29,30^

In addition, PHAs have been known to degrade effectively in aquatic and soil-based environments during both enzymatic and hydrolytic degradation.^10,30^ One recent study by Omura and colleagues examined a series of commercially available biodegradable and non-biodegradable plastic materials at different ocean depths, in both coastal and deep-sea floor environments, and PHA materials and copolymers were shown to decompose effectively with specific microorganisms.^10^ These findings have generated interest in developing PHA materials with potential to be decomposed as soon as they are discharged into the ocean, with many new and exciting opportunities for PHA development.^10^

Despite the considerable potential offered by PHAs towards enabling a transition away from conventional fossil fuel-based plastic, the existing industrial scale commercial production and distribution of these biopolymers has remained somewhat limited.^29,31,32^ At present, there are several enterprises that produce PHAs worldwide, including BioMatera (Canada), Biome Bioplastics (United Kingdom), Biomer (Germany), Cardia Bioplastics (Australia), Danimer Scientific (USA), Kaneka (Japan), Full Cycle Bioplastics (USA), Mango Materials (USA), Nafigate (Czech Republic), NaturePlast (France), Newlight Technologies (USA), Tianjin GreenBio Materials (China), Yield10 Bioscience (USA and Canada), and Tianan Biologic Material (China).^27,33,34^

Some of the current major limitations of PHA production on an industrial scale include availability of appropriate feedstocks, low productivity, accumulation, and yield, as well as a complex system of microorganism genetic engineering and biosynthesis culture setups.^32^ All of these factors have contributed to the high cost of commercially available PHAs. For instance, compared to the price of producing virgin plastic material in USD for PP ($0.45–0.68 per lb),^35^ PS ($0.54–0.82 per lb),^35^ PLA ($1.00–1.37 per lb),^36^ or other starch-based biodegradable polymers ($0.91–2.04 per lb),^35–37^ PHB on average costs between $1.81–3.20 per lb based on data collected between 2020–2023.^38,39^ Unfortunately, there have also been challenges for commercial production including overall setup, production, and capital costs, slow rate of market adoption, and limited projected financial returns leading to additional economic challenges for PHA biopolymer production.

Moreover, while the diversity of verified types of PHAs is extensive, the amount of commercially produced PHAs is rather limited, with available polymers and copolymers largely constrained to a narrow set of chemistries that are dominated by polyhydroxybutyrate (PHB), otherwise known as poly(3-hydroxybutyrate) (or P3HB). PHB the most commonly commercially available PHA.^27,31^ Other commonly available copolymers of PHB include poly(3-hydroxybutyrate-*co*-3-hydroxyvalerate) (typically written as PHBV or P3HB3HV) and poly(3-hydroxybutyrate-*co*-3-hydroxyhexanoate) (PHBHHx).^27,40,41^

The limited chemical diversity and tunability exhibited by commercially available PHAs also result in materials selection and design challenges.^42^ Specifically, the narrow and often unsuitable range of thermal processing windows and mechanical properties, and limited degradation rates which are sometimes comparable to other less expensive biopolymers such as polylactic acid (PLA) pose great hurdles for PHAs towards their use in a range of applications.^29,43^ Currently, this renders PHAs either unsuitable or too expensive to be realistic alternatives to traditional plastics.^20,44–46^

However, despite their expense, PHAs still offer many unique advantages in terms of biodegradability, compostability and non-toxicity, and potential for long-term sustainability in commercial use when compared to more widespread plastic alternatives available today.^47,48^ While these hurdles pose significant and interdependent challenges to widespread PHA use, they also present unique opportunities from both research and economic perspectives to understand and improve the overall PHA materials design paradigm for this class of materials as well as other commercially limited biopolymers.^16,19,23^

**Fig. 1:**
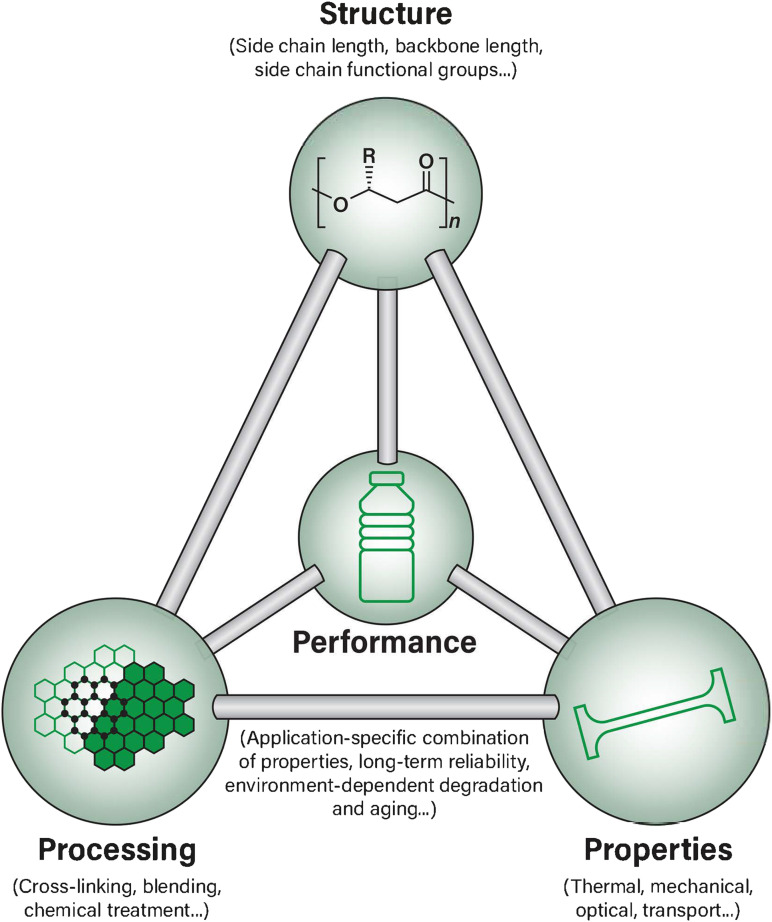
An overview of the materials tetrahedron and the relationships between each aspect as applied to PHA biopolymer material design.

When addressing materials design challenges such as those currently faced by PHAs, one central materials science framework relates the properties, structure, processing, and performance (PSPP) of a material in interconnected pair-wise relationships epitomized by the classic materials tetrahedron, as shown in Fig. 1. The concept of PSPP relationships has been extensively applied to many areas of materials science ranging from alloy design to perovskites and heterogeneous catalysis to pharmaceuticals, but remains relatively less explored in the context of bio-derived polymer design, in general, and for PHA-based biopolymer design, specifically.^49,50^

The materials tetrahedron has also been recently re-imagined in a contemporary, information science-focused perspective. This new perspective on a classic paradigm also aims to reduce the overall time and cost to discover and develop new materials by re-establishing the tetrahedron as a framework for novel design principles.^51^ It is well established that the PSPP relationships are particularly suitable for developing and examining quantifiable relationships between an available material's structure and its potential properties, as well as in connecting its processing history to the target performance.^51^ Therefore, there is potential to both explore and improve the scientific understanding of these relationships as well as enhance the commercial PHA design space by using a well-known PSPP framework and establishing it in an innovative way towards biopolymer identification, design, and development.

In traditional implementations of the materials science tetrahedron, the structure, property, processing, and performance parameters have been essential. Each of the branches of the tetrahedron provide a framework to not only understand their interconnected nature, but also has been historically used in the rational design and optimization of novel and existing materials in other areas of materials science.^51,52^ In this review, we have surveyed these PSPP relationships in the context of PHA biopolymers by examining current state-of-the-art examples of ongoing research.

Here, we apply this PSPP relationship framework to understand and highlight the scope of the underlying chemical space, outstanding design challenges, conflicting trends in properties, as well as current limitations and potential for future improvements in the broad research area of PHA biopolymers that have the potential to utilize modern experimental approaches. Our work also establishes a precedent for other types of biopolymers to be examined in a similar, materials-centric, design-focused way.

## 2 Control of PHA structure

### 2.1 Chemical and morphological structure of PHAs

At the most fundamental level, the chemical structure of a polymer is dictated by the atomic constituents forming the monomers and their connections. Monomers of one or more types can then combine in a backbone and side chain in different compositions to form either homopolymers (made up entirely of one monomer type) or copolymers (made up of several different monomers). For a given composition, the relative placement of different monomers along the polymer backbone can further give rise to different copolymer microstructures (for instance, random, block or alternate) resulting in qualitatively different interchain interactions. These interactions in turn dictate how closely the polymer chains are packed together, giving rise to the overall mesoscopic morphological structure.^53^

**Fig. 2:**
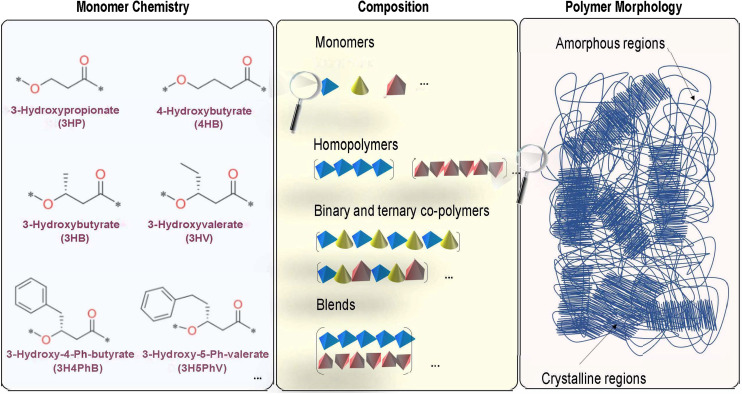
An overview of the chemistry of PHAs and a simplified breakdown of how these factors affect the structure by level of the material. Subsequent levels of composition variation are shown from the left panel to the right panel. The monomer chemistry panel (left) indicates changes that can be made to the monomer itself to distinguish between monomers of PHAs such as the number of carbons in the side chain and backbone. The composition panel (center) indicates changes that can be made to the building blocks of the polymer itself to distinguish between a homopolymer, a copolymer, variations of copolymers, and blends. The polymer morphology panel (right) indicates the next layer of changes that can be made to macro-scale regions of the polymer to produce variations between polymers of the same composition.

Therefore, the overall structure in polymers, including PHAs, which are in general linear, aliphatic polyesters, is largely governed by: (1) the intrinsic chemical nature of the building blocks at the atomic-scale, (2) non-bonding interchain interactions at the microscopic level, and (3) the relative ratio and distribution of crystalline and amorphous regions at the mesoscopic level to form the overall morphology of the polymer.^53–55^ These notions are graphically illustrated in Fig. 2. The wide range of dispersity indices and variations in molecular weight across PHAs produced biologically have also been shown to generate higher uncertainties in the resulting thermal and mechanical properties, which presents future opportunities for developing an understanding of these relationships in a more quantitative way.^56,57^

There is a significant amount of structural diversity that is currently available for PHAs at the monomer level, leading to a wide range of variety in possible interactions at both the microscopic level and in the overall morphological structure of the polymer.^27,57,58^ There are currently at least 150 structurally unique PHA monomers that have been verifiably identified and biosynthesized since 1995, leading to many possible combinations of potential polymers and copolymers of PHAs.^56,58–60^ However, in addition to these monomers, potentially there exist a number of other chemistries that might be accessible in the near-future either biologically or *via* chemical polymerization, or *via* grafting.^61^ Because the materials properties of the PHAs depend on the multi-scale structural aspects described above, this opens a tremendous opportunity in terms of tuning PHA structure to meet specific property portfolio requirements.^60^

Much of the diversity in the actual physical PHA chemical structure is derived from three main sources in varying the components of the polymer: (1) variety in the number of carbon atoms in the polymer backbone, (2) the number of carbons in the side chain branching from a backbone carbon atom, and (3) the presence/absence as well as chemical nature of any side-chain functional groups in the structure.^27,62^ In regard to the latter two diversity factors, the side chain of a PHA typically ranges from 1–16 carbons in length, and there is also a variety of functional groups, such as phenyl groups, with varying numbers of carbons in the side chain.^62–64^

The features that can be modified to produce the unique PHA monomers are the previously listed three components, that can be seen as examples in Fig. 2. The left panel of Fig. 2 indicates modifications that can be made of the standard hydroxybutyrate (HB) monomer. For instance, the addition of another carbon in the main backbone chain of the structure will change the PHA monomer from 3HB to 4HB, while the addition of another carbon in the side chain of the structure will change the PHA monomer from 3HB to 3HV. Thus, the number of carbons in both the backbone and side chain of the structure itself will affect the type of PHA monomer that is produced. Additionally, changing the functional group in the side chain of the structure will also affect the monomer: for instance, the inclusion of a phenyl group in the side chain of the structure will change the PHA monomer from 3HV to 3H4PhB. These changes in the monomer will also affect the type of polymer and copolymer blocks that may be synthesized.

There is a separate, alternative classification terminology system that is frequently employed to address different types of PHAs on the basis of the number of carbon atoms in the backbone and side chain itself. These classifications include a PHA with backbone and side chains of less than 5 carbons, short-chain length (scl), and those with the number of backbone and side chains between 6–14, medium-chain length (mcl), or even longer carbon chains, long-chain length (lcl).^40,64–66^ In addition, it is known that due to their method of biological synthesis and fermentation, PHAs formed by natural means are strictly isotactic and feature only (R)-configurations in their chiral stereocenter in the main carbon backbone of the polyester chain, but some variations have been synthesised by synthetic means.^27^

Each of these structural components have been demonstrated to have unique effects on the PHA polymer properties, affecting everything from its crystallinity to its thermo-mechanical properties and degradation rate. An example of this behavior can be observed in the case of polyhydroxyvalerate. This polymer is also written and abbreviated as poly(3-hydroxyvalerate), PHV, and P3HV. It may be referred to as HV in its monomer unit form. PHV is often also used as a copolymer with PHB. Although PHV contains just one additional carbon in the side chain compared to PHB, PHV has a lower melting and glass transition temperature and is far more elastic, in addition to varying the molecular weight of the copolymer *versus* the homopolymer.^67^ For instance, it has been shown that in general, a greater number of carbons in the side chain of the PHA leads to: a decrease in melting and glass transition temperatures, a decrease in elastic modulus, and an increase in yield at elongation, as well as potentially lower molecular weight depending on the method of synthesis, crystallinity, and polymer type.^68–70^

A greater number of carbons in the PHA backbone and side chain have been particularly associated with an increase in the overall degradation rate.^71^ This has largely been attributed to the increased number of carbons creating additional steric hindrance that the additional groups bring to the semi-crystalline packing structure of the PHA. A decrease in PHA crystallinity in polymers with a higher number of carbons in either the backbone or side chain and PHA copolymers has also been reported.^72^ Because these amorphous regions have enhanced susceptibility to hydrolytic degradation, this phenomenon of enhanced degradation is believed to be largely due to this decrease in crystallinity within these semi-crystalline polymers.^73,74^ These effects will be discussed in further detail in section 3 of this review.

In addition to PHA homopolymers and block or random copolymers combining multiple types of PHAs, there have been previous studies that have altered the structure of existing PHA polymers by incorporating other types of polymers in blends.^65,75–78^ Further modification of the polymer structure using additives, which in turn affects the properties of the resulting material, has also been extensively explored in prior investigations.^79–82^ These additions to PHA polymers are considered here as additional modifications to the base PHA structure, rather than a unique element of the structure of the PHA polymer itself. These modifications will be discussed in further detail in section 3 in this review. However, we note that the interconnected nature of the polymer chain structure has been shown to influence the material in all aspects of the materials tetrahedron, ranging from properties to the possible processing routes for the PHA.^83^ Additional details regarding the synthesis of PHAs as described in the following section, by both biological and chemical means, are critical to also consider when developing a more holistic PSPP relationship for these materials.

### 2.2 Factors affecting structure of PHAs in biosynthetic and chemical routes

There are many important factors affecting the structure of PHAs, ranging from the synthesis route to the processing method. PHAs are synthesized by a wide range of microorganisms as part of their natural metabolic processes as internal energy reserves in the presence of both: (1) excess levels of carbon and other energy sources (feedstocks such as wheat or flour, sugars (*e.g.*, glycerol), food waste, dairy processing waste, or cooking oil, carbon dioxide gas, other forms of starch, *etc*.) and (2) stressful environmental conditions, such as critical nutrient limitations (*e.g.*, nitrogen).^84–90^

PHAs are most widely produced by biological, natural synthesis processes within a living cell. Under normal conditions and a non-stressful environment, many PHA-producing microorganisms will utilize nutrients such as nitrogen, phosphorous, oxygen and carbon-rich organic matter in the tricarboxylic acid (TCA) or citric acid cycle to consume energy.^57,91^ The TCA cycle is a well-studied series of chemical reactions that occur within cellular organisms to release stored energy by oxidation of acetyl-CoA derived from energy sources such as fats and carbohydrates.^92^ Recently, extensive studies have been conducted specifically on the metabolic pathways for the biosynthesis of various natural and non-natural PHAs that explore these relationships from a mechanistic perspective.^28,57,61^ These reviews provide explicit detail on how PHA biosynthesis is conducted from the metabolism from the carbon source to the polymerization of monomeric components into specific PHAs and should be consulted for specific metabolic pathway and synthesis enzyme details.^28,57^

Both the available biochemical pathways and diversity of PHA-producing organisms have been well-documented in the literature.^93–97^ Under stressful conditions, such as excess carbon, or limited oxygen or nutrients, microorganisms and bacteria will metabolize the excess organic matter and use the acetyl-CoA normally used in the TCA cycle to instead synthesize PHA polymers as energy reserves using either existing or genetically-encoded biochemical pathways.^86,91,92^ Some examples of these biochemical reactions include hydrolysis, transesterification, and glycolysis, which are each also used to convert PHA precursors such as fatty acids, sugars, and amino acids into PHA monomers. These monomers are subsequently converted to intracellular granules of PHA polymers, which are stored in the microorganism.^98,99^ Depending on the available biochemical pathways and the substrate, both wild-type and genetically modified microorganisms have been shown to be able to synthesize a variety of PHAs.^45,97^

In general, PHA commercial production involves the culture of microorganisms for replication, followed by mixing in a cultivation tank or bioreactor with a substrate such as glycerol, starch, or other carbon sources using products such as carbon dioxide gas, food waste, dairy processing waste, and used cooking oil.^86,90,100^ Under specific, nutrient-limiting conditions, the microorganisms will then produce PHA polymers internally, consuming the substrate to varying degrees of generation of polymer mass.^80^ For instance, wild-type *C. necator* has been observed to generate up to 80% of its original dry weight in PHA polymer.^57,89^

The raw PHA polymer is released from the biomass *via* cell lysis following completion of biosynthesis, followed by solvent extraction.^101,102^ Lysis is often performed to break open the organism and perform the extraction using a centrifuge and a series of filtration steps. The PHA can then be purified and dried into a useable form, such as a powder or granule form for additional processing. These methods have been well-documented by a variety of both historical and current studies.^101,103^ The leftover residual non-PHA biomass can also potentially be used for other uses such as fertilizer.^89^ The extent to which commercial production methods of PHAs themselves directly and indirectly affect the resulting chemical structure is still an open area of research.^57,97^ Prior investigation has revealed that many possible PHA homopolymer and copolymer structures can be obtained by utilizing variations in the *microorganisms* themselves, indicating that one of the main contributing factors to producing different types of PHAs are the available metabolic pathways that are present within each unique organism.^104–108^

Recently, an extensive review was assembled on the diversity of microbial production of bio-derived monomers and the environmental circularity in their production, including individual strain, carbon sources, and yield. This also mirrors the diversity of PHA production by microorganisms and bacterial strains.^109^ There are a wide variety of PHA producers, which belong to many different types of phyla ranging from Mycobacterium and Streptomyces to Neisseria and Pseudomonas, with many studies articulating the necessary genetic and metabolic pathways for the different types of organisms to produce different types of PHAs.^57,102,110,111^

Currently, *C. necator*, *P. oleovorans*, *Pseudomonas* sp., *Synechococcus* sp. and *Halomonas* sp. are among the most commonly studied producers of PHB, with both wild-type and genetically modified organisms investigated extensively in previous studies.^57,83,91,110,112–114^ There is an emphasis in the literature to determine the optimal organism type, growth and carbon source conditions, and overall genetic pathways with scalable factors to produce the highest yield of cell dry mass as possible.^111,115,116^ For instance, one recent study explored the production of a copolymer of 3-hdyroxydecanoate and 3-hydroxydodecanoate using *Pseudomonas aeruginosa* MC5300 with yields up to 75.6% of original dry weight of the cell with up to 16 hours of growth.^111^

The *carbon source and nutrient limiting conditions* that fuel biosynthesis pathways within PHA-producing microorganisms are also required contributors to the synthesis and polymerization processes performed by the organism, and greatly influence the extent to which the structure and chemistry of the PHA may be manipulated.^102,112,117^ Without a carbon source, the organism would have no way to utilize their unique enzymes and biochemical synthesis pathways to produce a PHA polymer.^34,62,118^ Here, we distinguish between carbon sources supplied to the organism as a substrate, and nutrients. Nutrients, in contrast to carbon sources, are other molecules in the cultivation or fermentation broth supplied to the organism to help it to grow, and may include vitamins, co-factors, nitrogen, phosphorous, or others – depending on the organism.^28,57,61,102^

Therefore, although how each organism utilizes these substrates is a unique process according to their own biochemistry, the carbon source and nutrient types themselves are just as vital, and may affect the resulting material.^28,102,119^ The effects that each of these factors have independently as well as in tandem with one another are not currently well understood although well-documented. This opportunity presents a potential new research direction for a large-scale experiment quantifying the relationship of carbon source and nutrient type to the outcome of the PHA produced by the biosynthetic pathway.

A variety of carbon sources have been tested in laboratory-scale bioreactors ranging from pure carbon dioxide and methane gases to coffee, rice, vegetable compost, cooking oil, dairy processing waste, and glycerol wastes.^90,102,120^ Each combination of substrate and organism was shown to yield slightly different final PHA polymer side-chain lengths.^45,90,120^ Currently, Gahlawat has reported studies that demonstrate control over the number of carbons in both the backbone and side chain of the PHA and the ability to incorporate phenyl and other bulk side chain functional groups.^102^ Many studies have also achieved tremendous advances in genetic and metabolic engineering of the organisms to produce these specific chemical structures, and reducing the cost of required carbon sources and substrates.^120–122^

Previous studies have outlined the challenges with increasing the overall yield and structural diversity in PHA production.^100,123,124^ A recent review by Corti Monzon and colleagues has also surveyed the use of petrochemical hydrocarbons as potential feedstocks for PHA biosynthesis.^115^ Several of these studies have explored methane, octane, diesel, and also styrene from pyrolysis of PS, essentially utilizing feedstocks derived from the products of burning virgin petrochemical plastic waste, although notable scaling challenges have been reported.

Additional ongoing challenges to biological production of PHAs that remain to be addressed include integrating the relationship between the nutrient limitation and substrate with the microorganism type.^121,122^ The most common and prominent PHAs that have been commercially developed as of now include PHB, and copolymers such as PHBV and PHBHHx, as briefly discussed above.^27,31,40^ The functional groups that are most commonly accessed in current research include carbon side chains of varying lengths and phenyl groups.^27,83,112^ The ongoing challenge to produce optimal yields is continuously balanced with optimizing the recovery process with cost efficiency. Also equally important is the task of integrating PHA biosynthesis into existing production lines to increase PHA compatibility with existing polymer processing equipment.^83,86,100,125^

Combining more than one type of microorganism in an effort to produce higher yields of PHAs has been also extensively studied in a method known as mixed microbial consortia as an alternative to genetic engineering.^126^ This area of research focuses on engineering the environmental conditions, nutrient sources and substrates, and carbon sources, rather than tuning the genome of the microorganisms.^126^ However, the type, quantity, and distribution of the PHA structure produced by the consortium has been shown to depend on the nature of the substrate fed to the organisms during PHA synthesis, leading to the additional importance of the substrate to the overall biosynthesis path to creating a final PHA structure.^83,86^

Some recent studies have been able to utilize bacterial colonization directing techniques to improve the overall properties and performance in PHA materials. In a specific case, Campano and colleagues have explored the control of the bacterial colonization process using bacterial cellulose to produce PHB.^127^ Following acid treatment to the PHB-cellulose bonded films, the elastic modulus increased and oxygen permeability decreased with respect to the control PET film over 4 and 3 fold times, respectively.^127^

In contrast to biological synthesis, there is also an active area of research investigating the chemical synthesis methods of PHA production. The chemical routes of PHA synthesis by artificial (*i.e.* non-biological means) is an evolving field, and many reports specifically examine the potential chemical procedures by which to develop PHAs artificially or semi-artificially. For instance, one ‘chemical’ or semi-artificial means of PHA production involves the recycling of fossil fuel-based feedstock or other chemical waste products to provide the energy resources for bacterial to generate the PHB. In this way, these approaches are not necessarily ‘chemical’ in nature as they still involve the biological synthesis of PHAs, but they incorporate chemical feedstocks into the biological means of developing the polymer. This process of ‘up-cycling’ waste feedstocks has been well-explored.^86,97,128,129^ One approach by Parodi and colleagues has also incorporated waste PHB itself directly into the feedstock cycle to synthesis ‘new’ PHB material, working to complete the circular waste-to-use lifecycle using an Oxa-Michael reaction to generate PHB monomers.^130^ A recent study by Zhang and colleagues has exemplified this ‘hybrid’ synthesis approach to combine both chemical and biological processes to produce PHAs.^131^ These approaches are typically less-utilized than conventional biological fermentation processes.^27,58^

Other approaches to developing PHAs by chemical means have explored alternate routes to modifying, by blending or grafting for instance, or chemically synthesizing the polymer. For instance, Olivera and colleagues have reported chemical modification of PHAs *via* halogenation, epoxidation, hydroxylation, carboxylation, chemical cross linking *via* sulphur vulcanization, peroxidation, or radiation-induction, resulting in altered properties of the source biological material.^132^ In contrast, chemical synthesis of aromatic PHAs and aromatic-aliphatic PHAs has been achieved by Westlie and Chen *via* stereoselective ring-opening polymerization of eight-membered cyclic diolides.^133^ This approach resulted in high molecular weight copolymerized PHAs, over 200 kg mol^−1^, which are mechanically resilient, hard, but ductile, up to 191% elongation at break. In addition, these copolymers of PHB also achieved a thermal decomposition temperature of 281 °C, which is much higher than typically biologically produced PHAs.^133^

Another commonly-studied starting pathway for developing synthetic PHB by chemical means features alternating propylene oxide and carbon monoxide. This is typically a challenging retrosynthesis and copolymerization process. In addition, ring-opening polymerization of β-lactones is also used. Both of these processes require additional development for full industrial scaling.^27^ It is well-established that the conversion of different substances to PHA polymers by both small-batch and industrial biological means is a result of selectivity, genetic pathways, nutrient limitation, carbon sources, and biocatalysts and enzyme availability, all of which can lead to high dispersity indices and high degrees of variation in the polymer molecular weight as well as batch to batch differences in the length of the polymer chains.^27,58^ These reports indicate chemical modification or production of PHAs can lead to customizable property value ranges and yield exciting new opportunities for further study.

The effects of stereochemistry of chemically synthesized PHB with regard to the crystallization behavior have been recently studied by Caputo and colleagues, in foundational work in the chemical synthesis of PHB.^134^ Detailed information regarding both the chemical synthesis and characterization of PHB polymers of different molecular weights were reported, including information regarding the stereochemical mixtures of synthetic samples as well as the growth rates of spherulites in PHB of varying molecular weights ranging from 9000 kg mol^−1^ up to 120 000 kg mol^−1^. The study also reported valuable information regarding the relationship between crystallization time, crystallization temperature, and molecular weight for synthesized PHB. High molecular weight PHB requires more time to crystallize during cooling, resulting in low values of crystallization temperature and the lowest degrees of crystallinity among the tested samples.^134^

These examples demonstrate how a change in structure can affect other PSPP relationships, such as the properties of PHAs, which are described in the following section. In general, the qualitative trends of the chemical, copolymer sequences, and morphological structure and their effects on various PHA properties are known to some extent given monomer chemistry and composition. However, a fully comprehensive quantitative structure–property relationship for PHAs relating their chemical synthesis to their structural characteristics and performance over a wide range of chemistries have not yet been fully established. This represents an exciting area of future research.

## 3 Properties of PHAs

### 3.1 Overview of PHA materials properties

When investigating the properties of PHAs, researchers must inherently bring the interconnected nature of PSPP relationships for PHAs as a complex biopolymer into consideration. As a candidate alternative to conventional plastics, the biocompatibility, degradation rate, thermal processing and melting temperature ranges, elasticity, mechanical strength and durability, and molecular permeability rates are all properties of interest in different established applications.^1^ In addition, the optical properties of PHAs, such as chirality, opacity or transparency, may also be considerations depending on the specific target performance demands.^102,135^ PHAs are highly biocompatible and exhibit fully degradable and compostable (even in non-industrial composting facilities) behavior across a variety of PHA types and in different environments, both environmental and biological.^1,17,100,136–138^ PHA degradation products are completely non-toxic, and include water and carbon dioxide if oxygen is present or other molecules under anaerobic conditions.^1,102^

Recent reviews have extensively detailed the properties of PHA-based homopolymers and copolymers, including their biodegradability and degradation rates as reported in experimental literature, particularly in comparison to fossil fuel-derived polymers.^15,69,83^ Here, we place these surveyed properties of PHAs in relationship to their structure, processing, and potential performance within the framework of the materials tetrahedron. Efforts to achieve the balance of these property targets have made the landscape of PHA research a challenging and exciting field.

### 3.2 Thermal properties of PHAs

**Fig. 3:**
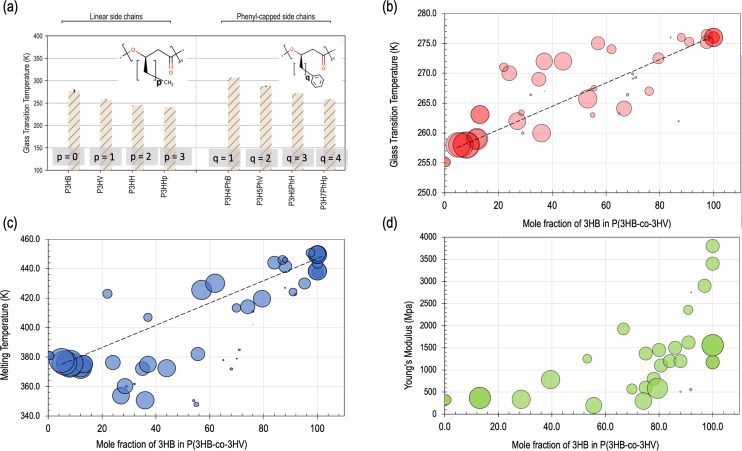
An overview of variations in specific materials properties with respect to changes in structure, mole fraction, and molecular weight. (a) Plot showing changes in glass transition temperature with increasing number of carbons in both linear (left) and phenyl-capped (right) side chains. PHAs are displayed on the horizontal axis and increasing number of carbons in the side chain are indicated from left to right. Reproduced with changes from ref. 139 with permission from the Royal Society of Chemistry: Physical Chemistry Chemical Physics publishers, copyright 2020.^139^ (b) The relationship of glass transition temperature to mole fraction of 3-hydroxybutyrate (3HB or HB) in PHBV. (c) The relationship of melting temperature to mole fraction of HB in PHBV. (d) Plot demonstrating the relationship of Young's Modulus to mole fraction of HB in PHBV. In panels (b–d), increased size of circular data point indicates an increase in molecular weight. The data for panels (b–d) has been adapted from the Royal Society of Chemistry publishers, copyright 2020.^139^

The thermal property ranges of PHAs showcase the interconnected nature of the materials tetrahedron for PHAs. Specifically, the glass transition and melting temperatures of PHAs are two well-investigated properties that are critical for compatibility with existing polymer processing equipment, long-term scalability, and ultimately, commercial viability.^45,86^ However, thermal properties have also been previously shown to be a hurdle for scalable manufacturing of PHAs.^140^ Three of the most important established and thoroughly-studied properties of PHAs are summarized in Table 1 as well as in Fig. 3, that include glass transition and melting temperatures along with mechanical properties such as elastic modulus (*E*) that will be further discussed in the mechanical properties discussion later in this section.^1,45^

**Table 1:** Thermomechanical properties of PHAs compared with selected conventional polymers. The selected seven fossil fuel-based commodity plastics accounted for 75.1% of Europe's yearly plastic production in 2020.^153^ Elongation at break here is the same as strain at break (%). Property values are listed in a range of averages over the entries in the literature sampled and the PolyInfo repository at standard conditions for available materials

Polymer	*T*_g_ (°C)	*T*_m_ (°C)	Elongation (Strain) at Break (%)	Young's Modulus (MPa)	Ref.
**Biopolymers**					
PHB	3–15	160–182	2–8	1100–4000	1, 56, 70, 148, 150 and 155–158
P4HB	[−51, −47]	53–61	697–1000	104–181	1, 27, 56 and 159–161
P(HB-*co*-20%HV)	[−6, −1]	100–145	20–50	700–2900	1, 56, 70, 150, 155, 156 and 162
P(HB-*co*-26%HP)	[−20, −8.1]	77–85	634–650	227–300	1, 163 and 164
P(HB-*co*-10%HHx)	[−7, 6]	127–160	240–400	500–900	1, 56, 72, 144, 152, 155, 161, 165 and 166
P(HB-*co*-17%HHx)	[−2, 6]	120–147	50–200	160–300	1, 56, 72, 144, 152, 155, 161, 165 and 166
P(HB-*co*-47%HV-*co*-16%4HV-*co*-)15%HHx-*co*-2%HO	[−15, −5.5]	118–155	1000–1089	110–390	1, 70, 72, 144, 152, 155, 161, 165 and 166
P(HO-*co*-36%DO)	[−44.5, −4]	126–153	40–70	190–220	1, 69, 70, 153, 155 and 167
PLA	50–80	130–200	7–20	1200–2700	43, 162 and 168–170
PCL	[−67, −60]	56–60	120–1000	15–400	1, 70, 162 and 168–171
**Conventional polymers**					
LDPE	[−100, −36]	88–130	300–600	200–670	1, 43, 70, 162 and 168
PP	[−20, −10]	160–176	50–160	1600–1950	1, 43, 70, 162 and 168
PET	67–81	253–260	65–230	2200–9350	43, 70, 162 and 168
PVC	80–87	100–260	12–32	300–2400	70, 162 and 168
PEN	100–120	268–270	42–50	106–2310	70, 162 and 168
PS	98–100	210–255	2–4	1600–3100	70, 162 and 168
Nylon-6,6	47–70	220–570	61–110	1300–4200	70, 162 and 168

In general, PHAs have melting points comparable to other well-known polymers such as PLA and polycaprolactone (PCL). PCL is well-established as a petrochemical-derived polymer that is biodegradable. PLA is also established as a bio-based polymer that is compostable in industrial facilities. However, the melting points of PHAs in general are typically lower compared to more conventional polymers such as PET, PS, or Nylon-6,6, as a result of their structure.^141^ PHAs also have generally low glass transition temperatures, for instance compared to PLA (but still higher than that of PCL), as shown in Table 1.^135,142^ PHB is usually highly crystalline at room temperature: up to 95% in some reports, but commonly observed with a degree of crystallinity closer to the 50–80% range.^27^

Inherently, PHB also has a narrow melting temperature range of 160–180 °C, but typically close to 170 °C within the range of the melting temperature of PLA and higher than that of PCL, with a low glass transition temperature between 3–15 °C.^1,27,27^ Changes with respect to these values based on structure and composition changes are visually depicted in Fig. 3, particularly in panels a–c. It has been noted that significant degradation and molecular weight loss occurs when processing PHB above its melting point, that leads to difficulties using PHAs with conventional methods and equipment such as hot presses or extruders.^40,143–145^ A comprehensive quantitative relationship between the copolymer ratio and both the thermal and mechanical properties in isolation have been previously established and prepare a framework for a more holistic understanding of the PSPP link between structure and resulting thermal properties.^77,102,141,146^

Adjusting side chain length and copolymer ratios is a balancing act for the desired application for the PHA to manage the properties and processing compatibility of the material. The design tradeoff is often applicable also in the case of overall biocompatability. A recent literature survey by Turco and colleagues has explored how the flexibility and processability as well as thermal stability of PHB can be tuned by adjusting PHB-PHBV copolymer ratios.^147^ For instance, differences in the size of the repeat unit (scl *vs.* mcl PHAs) were shown to affect both the crystallinity and the glass transition temperatures of copolymer PHAs. In the case of PHB-mPHA blends, the review explored how longer chain PHAs were more likely to have lower melting temperatures. In addition, the longer chain PHAs were also shown to exhibit more amorphous regions and faster overall hydrolytic degradation rates. This was explained to be due to the amorphous regions being more susceptible regions of a semicrystalline polymer to hydrolytic attack and subsequent degradation.^147^

Copolymerization of PHB with other PHA materials, across the short, medium, and long-chain length scales can significantly alter the thermal properties of the overall material. Kim and colleagues have recently reported double melting peaks on differential scanning calorimetry (DSC) scans of PHBV copolymers when assessing the melting point and crystallization properties of the polymers for an enzymatic degradation study.^148^ The splitting of the peak was linked to the two distinct melting temperatures for the PHBV copolymer. The authors discussed how previous investigations have reported differences in crystallite lamellae thicknesses between PHB and PHV, leading to distinct phase separations in the crystalline ordering of the PHBV overall copolymer.^148^

Specifically, long PHA polymer chains that have well-ordered, thicker lamellae, and high crystallinity up to 80% – such as monomeric PHB – have demonstrated higher melting temperatures, whereas PHV with its sterically-hindering additional numbers of carbons in the side chain leads to less well-ordered chains with thinner lamellae and lower melting temperatures.^148^ This observation in the distinct melting temperatures of PHA copolymers that can be adjusted by copolymer ratios can be further explored and exploited to tune the overall thermal properties of copolymerized PHAs for specific temperature ranges.

Either by copolymerization or blending with other additives, adjusting the copolymer structure of PHAs can significantly improve thermal stability while maintaining both the degradability and mechanical properties. In terms of adjusting PHA-PHA copolymer ratios to affect the properties, generally increasing HB content can increase the glass transition and melting temperatures that generally enhance the strength, while increasing the HV content increases the thermal processability,^56,149^ as shown in Fig. 3. This trend contributes to its performance in many applications, and has been shown to also affect the overall degradation rate in both hydrolytic and enzymatic degradation environments.^149^

Recent reports have shown that by working with additives such as starch or lignin, or grafting and block copolymerization with other biopolymers such as PCL, polyethylene glycol (PEG), polymethylmethacrylate (PMMA), and PLA, more expansive results can be achieved to tune the thermal property ranges in tandem with the degradation rate,^26^ biocompatibility,^149^ and mechanical strength.^43,75^ However, further detailed research involving structural analyses coupled with degradation studies may be necessary in evaluating the weight-to-polymer breakdown ratio in these PHA blended materials to ensure that the overall material is fully degrading, rather than just the PHA components degrading alone. It is well-established that the molecular weight,^150^ dispersity,^151^ and overall chemical composition^152^ have all also been shown to affect the thermal properties of polyesters and PHAs specifically.

### 3.3 Mechanical properties of PHAs

The mechanical strength and durability of PHAs are very important for the overall applicability of PHAs as general commodity plastic alternatives or for biomedical applications to be able to meet the physical and structural integrity demands of the desired application. These diverse applications and property value requirement ranges may vary from disposable food packaging and single-use cutlery needs to bioresorbable sutures and hard tissue regeneration scaffolding.^1^ With an ultimate goal of tuning the mechanical properties of PHAs for them to serve as acceptable alternatives to other less compostable or less biocompatible materials such as PLA, PS, PVC, or LDPE, it is important to be aware both of the structural changes as these property values are targeted as well as their ability to meet intended performance value ranges.^56,100^

The mechanical property ranges of PHAs further demonstrate the complex structural dependence of the material and the need for PSPP-relationship focused property tuning to meet specific performance demands. Depending on the length and composition of the monomer units, PHAs have demonstrated a broad spectrum of mechanical properties. These can range from extremely soft, amorphous, and ductile mcl- and lcl-type PHAs such as poly(3-hydroxyoctanoate) (PHO) or poly(3-hydroxydodecanoate) (PHD) to the brittle and crystalline scl-type PHAs such as PHB or the very soft, yet still scl-type PHV.^1,171^

Due to its highly crystalline linear structure, PHB has not only a low glass transition temperature and narrow melting temperature window, but it is also highly brittle and stiff compared to other thermoplastics such as LDPE, and behaves mechanically more on the order of isotactic PP, PS or PET, with lower strain rates at failure.^140,141,171^ It is also more hydrophilic than PET, resulting in higher water absorption, that can be utilized in tandem with its inherent mechanical properties for changing the structural integrity of the PHB product.^147^ Discussion on the effects of crystallization on the mechanical properties of PHBV copolymers have proposed that the amorphous polymer chains are restricted by two subsequent crystallization events after solidification from melt processing.^1,82^ These effects were found to be mitigated through the addition of nucleation agents to reduce the cracks that form in the brittle crystalline structure of PHB spherulites created during the melt processing stages.^1^

Commonly used measures of mechanical properties that have been established in the literature for PHAs include tensile strength at break (*σ*_b_), tensile strength at yield (*σ*_y_) and elongation at break (*ε*_b_).^45,171^Table 1 provides a quantitative comparison of common PHAs along with a comparison to conventional polymers as a reference to their mechanical properties in addition to their thermal properties. The mechanical strength and durability of PHAs tend to be either comparable, or on the lower end of the performance range, when compared to more established polymers such as PLA and PCL.^43,140,172^

Comparisons are often made between PHAs, as listed in Table 1, and PLA, which itself is a typically glassy and strong but brittle polymer (Young's modulus around 1200–2700 MPa and elongation at break around 7–20%) or more conventional polymers such as PP, with a Young's modulus and elongation at break falling within the ranges of approximately 1600–1950 MPa and 50–160%, respectively.^173–175^ For employing PSPP-focused co-design principles, the idea that changing the properties of PHAs should be studied not just in isolation but in tandem with their structural features, both thermal and mechanical properties, and future processing conditions is paramount.

For instance, blending and copolymerization with other materials is a widely used approach to achieve both broader thermal property and mechanical property ranges. These properties are significantly altered with increasing copolymer ratios such as increasing HV content.^1,147^ The crystallinity of PHBV copolymers has been shown to decrease with increasing HV content, which in turn leads to decreased melting and glass transitions temperatures, increased strain at break, and reductions in elastic modulus.^1^ PHBV also has a high elongation at break, in the range of 45–1080% with increasing HV copolymer ratio.^109^ Increasing number of carbons in monomer building blocks has been demonstrated to significantly affect the mechanical properties of PHA copolymers. In one recent report by Sohn and colleagues, the HHx monomer was shown to reduce the melting temperature of the copolymer while increasing the elongation at break.^109^ The resulting polymer demonstrates improved strength and flexibility through the incorporation of HHx monomers.^109^

It is important to highlight here that every change in PHA properties due to copolymerization or blending in order to yield a desired final property range inherently builds in downstream processing compatibility changes, such as decreases in viscosity and molecular weight under extrusion.^171,176^ The addition of non-PHA polymer chemistries has also can change the properties of hybrid PHA blends. These changes can in turn affect the processability and potentially also alter the degradation rate of the PHA, making it more or less degradable. Examples of other types of natural materials incorporated into PHA blends include corn starch, wood flour, glucose, cellulose or nanocellulose, nanoclay or nanominerals.^75,76,79,83,177,178^ Blending of PHAs with other polymers such as PCL and PLA will be discussed later in this review in section 4.2.

As a general trend, across structural and copolymer changes in both scl- and mcl-PHA types, there is a distinct tradeoff between the strength of the PHA and its ductility.^45,149^ Blending can increase the elastic modulus of PHA materials while maintaining high levels of elongation at break, such as for P(HO-*co*-DO) and P(HB-*co*-3HHx), the latter of which has been shown to maintain an elastic modulus comparable to some types of PVC, while maintaining a much higher elongation at break and lower glass transition temperature (Table 1). The structural differences in these blends, grafts, and block copolymerization with other polymers (including other PHAs) has not been demonstrated to alter the physical structure of the PHA, but rather the more mechanically durable block or component of the polymer may end up bearing more of the mechanical stress during shear, resulting in a strong and also degradable material.^65,100,179,180^ PHA blends are discussed in further detail in section 3 of this review.

There is also a high degree of variability in the mechanical properties of PHAs as a function of crystallinity. This is a very important structural characteristic of the material and can be altered in a circular manner by the processing, and potential recycling of degraded PHA for feedstock in new PHA materials.^26^ Not only has the crystallinity been shown to affect the strength and durability of the PHA, but previous work has shown that % crystallinity of the material also impacts the degradation rate.^100,179^ The mechanical properties have also been shown to change with respect to crystallinity, with a higher percent crystallinity being associated with more brittle behavior (higher elastic modulus, lower percent elongation at break) and *vice versa*.^172,181,182^

These relationships between the properties of PHAs and the crystallinity of the material are still being explored. For instance, one review by Kalia and colleagues describes in detail the use of additives such as nucleating agents to increase crystallization, stabilizing agents such as antioxidants, and plasticizers such as glycerol and sorbitol to improve the mechanical strength of PHAs while retaining its original degradation rate.^86^ This work has helped establish the exploration of the properties of PHAs with consideration to crystallinity as an open area for future research.^86^

### 3.4 Gas permeability of PHAs

The importance of the interconnected nature of PSPP relationships in PHA design are also highlighted by the molecular migration rates, water solubility, and diffusion barrier properties. These properties are critical components of PHA biodegradability, particularly relating their susceptibility to hydrolytic degradation and environmental factors such as humidity, moisture, and other conditions.^4,48^

Studies of the barrier properties of PHAs are of particular importance. Due to their non-toxic degradation products, PHAs have a unique advantage to seal food and other consumer goods in strong barriers without the need for long-term degradation rates, industrial composting facilities, and without harmful migration of decomposition products. Studies have reported these trends for PHAs while the materials are able to maintain similar water permeability values to traditional plastics such as PVC and PET.^4,135,142,183^ The full molecular permeability and migration rates of water vapor, oxygen, and carbon dioxide have been previously reviewed in depth by Naser and colleagues, as well as other literature surveys and repositories.^1,4,102^ However, there remains more to be explored in future work from the PSPP design perspective for PHAs to fully articulate and quantify connections between their structure and performance.

The nascent commercial space for reliable and environmentally-friendly single use food packaging and wrapping is increasing.^70,183^ Some essential properties of these barriers include low oxygen and carbon dioxide permeability, low water solubility, and strong resistance to moisture to prevent spoilage and exposure to bacteria.^157,184^ For fresh fruit and vegetables that require respiration as part of their storage, bio-derived film plastics have been shown to have a higher molecular migration rate than commercial plastics films, making them uniquely advantageous by removing the anaerobic environment that seals foods when conventional plastic wrap is used.^185^

PHAs in particular possess relatively low oxygen, nitrogen, and carbon dioxide permeability rates. PHAs also generally display high methanol and water vapor permeability rates.^113,135^ In general, the semicrystalline chemical structures of PHAs, and PHB specifically, are hydrophobic, one of the reported rationales for their strong barrier properties. The HB and HV components of PHBV copolymers have been observed to have similar hydrophobicity values overall.^1,4^ It has also been shown that as HV content increases, there is a resulting decrease in crystallinity in PHBV copolymers.^185^

In addition, PHAs have the ability to out-perform other bio-derived materials such as PLA in protecting the desired product from environmental elements while not compromising on overall degradability.^135,142^ The water vapor permeability rates of both PHB and PHBV, up to 50 wt%, have been reported to fall within the range of PS and PVC, with oxygen and carbon dioxide permeability rates far below that of PP, PS, and PVC.^4^ One report by Allison and colleagues demonstrated that in the case of water, the activation energy of films of PHB was similar to those presented by other semicrystalline, traditional polymers such as isotactic PP, and the high value suggested the existence of positive interactions between water molecules and the polymer matrix itself.^23^ For other permeating molecules such as ethanol, propanol, and methanol, it has been shown that the permeability for organic vapors decreased as the size of the molecules increased.^23^

PHAs have been shown to be completely biodegradable in all environments, including home compost, septic systems, and municipal solid waste systems, without significant change to their permeability properties depending on environmental conditions.^135,157^ In one test under ASTM D5511 and 37 °C, 70–100% biodegradation of PHBV was achieved relative to the cellulose control.^177^ These properties have been discussed to arise from the structural and surface property differences in PHAs compared to other commonly used biopolymers such as PLA. Potential future studies involving modification of the inherent properties of the polymer indicate that this is an active area of ongoing research which can explore many exciting possibilities relating the structure of the PHA to its gas and moisture barrier properties.^186,187^

Future work in this area must also seek to understand how the barrier properties of PHAs and copolymerized PHAs with other materials may change with long-term storage or shelf-life degradation, and further quantify how these materials also change with storage at different temperatures beyond room temperature. For instance, how the barrier properties of these materials will change after storage in a hot car, or in the freezer for an extended period of time to ensure the efficacy and safety of products at different storage temperatures. These consumer-use questions are of particular importance to the commercial development of PHAs as products, but also lead to significant interest for better understanding the structural changes taking place in these different temperatures for real-world use and practicality and how they affect the barrier properties.

From both functional and consumer aesthetic perspectives, materials serving as films employed as packaging materials are expected to possess both UV protection and high transparency, in addition to their strong barrier properties.^188^ In one recent study, Ferri and colleagues blended PHBV copolymer with hydrophilic additives, up to 10 wt% tannin concentration, using solvent casting to prepare films to be stable at shelf, refrigerator, and freezer temperatures.^188^ These films were observed to have elastic modulus and tensile strength values within the same ranges as PLA and PS, and while increasing tannin content was found to lower film transparency, a compromise between transparency and UV protection was achieved at an intermediate concentration of tannins.^188^ The antioxidant activities of the blended polymers were also improved in addition to a decrease in carbon dioxide and oxygen permeability. Finally, the PHBV/tannin blend could also determine ammonia vapor using a color-change indicator that was investigated for its potential to determine concentration of ammonia as a food spoilage indicator.^188^

Previous discussion of how permeation of small molecules can only proceed into the amorphous regions of a polymer has lead to some interesting design perspectives. Modifications to the crystallinity or the free volume of the polymer can also modify the diffusion paths for the small molecules. In a study by Genovesi and colleagues, blends of PHB copolymers, including PHBHHx and poly-3-hydroxybutyrate-*co*-4-hydroxybutyrate (PHB-4HB), were examined in extruded, cast films. The study was organized to compare the increasing percentage of PHB-4HB to monomeric PHBHHx in terms of the oxygen, carbon dioxide, and water vapor permeability rates.^185^ The increased percentage of amorphous PHB-4HB copolymer in the PHBHHx blend contributed an increased oxygen permeability rate.^185^ While improving the mechanical properties of films or altering the thermal processing range of PHAs, significant changes to the gas permeability rates may also occur, and they must be taken into consideration when optimizing the performance of these materials.

These findings are important to establishing a relationship between the structural elements of PHAs. This relationship affects many interconnected property and performance metrics from a PSPP co-design perspective. Future studies will continue to explore these established relationships, particularly focusing on relating these rates in a quantitative way to the overall degradation rate of the material for long-term shelf life stability.

The full suite of structure–property relationships relating side chain length, polymer backbone, chemical nature of functional groups, morphology and stereochemistry to their physical properties is still a relatively new concept in biopolymer design. In particular, connecting these elements together for bio-derived polymers and understanding the PSPP relationships in the specific context of PHA design establishes a systematic approach for potential future studies. Further structure–property related research investigating their tunable physical properties, such as thermal and mechanical performance, surface features, crystallinity, amphiphilicity, and degradation rates is required to investigate the full potential for the PSPP co-design space.

### 3.5 Structure–property relationship exploration using machine learning

Although most widely available PHAs (such as PHB) do not inherently have a favorable combination of mechanical strength and flexibility desired for certain applications (such as consumer goods and food packaging), the possible compositions and copolymer sequence spanned by homo- and copolymers of PHAs in combination with other types of materials present a truly vast chemical space for future study. Not only does this paradox represent tremendous potential for improving these materials and tailoring their properties for the desired applications, but it also provides an exciting model material for new computational methods examining the design and identification process overall.

Identifying and designing PHAs with an optimal combination of application-specific properties is a daunting challenge using traditional trial-and-error based methods, given the diversity of the PHA family.^189–192^ The burgeoning field of polymer informatics presents new exciting opportunities in this direction. In particular, the use of modern data- and information-centric approaches has already led to exciting new opportunities for materials candidate screening.^191,193,194^ First principles-based traditional polymer design experiments involve challenging and resource-intensive property optimization experiments during the PHA design process to determine exact ratios of copolymers of PHAs for desired applications and testing these novel materials individually.^191,195,196^

Fortunately, as a result of the advent of the data-enabled paradigm, researchers now have increased access to larger-scale, quantitative polymer data. This data has been collected from individual real-world historical experimental findings reported in the literature and other sources such as computationally generated simulations of property data with uncertainty values.^197–199^ These resources include such open-access databases as the Materials Genome Initiative, PolyInfo, Polymer Genome, PolyBERT, and Khazana Computational Materials Database projects.^200–202^

Efforts such as the Polymer Genome project, in addition to serving as data repositories, also involve pedagogical efforts in data curation, representation design strategies, and a user-interface to navigate the data usage and prediction processes.^191,191^ Other efforts such as PolyBERT have recently been developed to include a polymer fingerprinting capability for chemical structures, using Natural Language Processing techniques to understand and interpret the structures as a language itself. This recent project maps the chemical structures of user-interactive fingerprints to real-world properties and can be used for scalable architectures including cloud infrastructures in an exciting new platform.^200^

Using machine learning (ML) and artificial intelligence (AI) methods, the tools and techniques made available within the field of polymer informatics enable development of surrogate polymer property prediction models.^189,193,199^ Efforts to develop ML-enabled methods of polymer property prediction and polymer design strategies have included automated extraction of insights and chemical knowledge from data and design of novel chemistries with a targeted set of properties in several foundational studies.^190,194,195,203,204^

For instance, many of the structural, chemical, and physical features of the polymer may be organized into a ‘polymer fingerprint’^194^ which relates unique information about the polymer identity into a ML-readable format. Features such as electrical conductivity, heat capacity, melting temperature, lattice parameters, band gap, and more^194,204^ can be used with landmark ML techniques such as Bayesian optimization in combination with models such as random forest regression and Gaussian process regression that can handle both small and large sets of non-linear, noisy data to predict or screen for more than one property.^190,195,205,206^ The efforts in this area of conventional polymer property prediction have seen an unprecedented activity and a number of successes in recent years, with a diverse set of applications ranging from solvent selection^207^ polymer dielectrics design for high energy density capacitors,^205,208,208,209^ polymer membrane design for gas permeability and separation,^187^ polymer fuel cells and sustainable energy storage applications,^209^ and polymer electrolytes design for Li-ion batteries to conducting polymers design for flexible electronic applications.^209,210^

ML-based efforts in this area necessitate the development of datasets that focuses on unique fingerprinting schemes for the polymers of interest. These studies work to capture the detailed atomistic and structural characteristics of the polymer in the dataset into encoded features that can then be utilized by the ML model to generate predictions. Using these unique features of the polymers, predictive models are generated to map the features to their resulting properties. This approach has been demonstrated in foundational work by Huan and colleagues^195^ and by Pilania and colleagues for polymer dielectrics.^190,206^

In another example, Chandrasekaran and colleagues used at least four levels of descriptors for the polymer fingerprinting in their report for conventional polymers. These details range from atomistic and quantitative-structure–property (QSPR) descriptors to morphological and higher-length scale descriptors.^202^ Many different categories of polymers other than PHAs were investigated in this study, including PVC, polyurea, poly(acrylates), and various forms of poly(ethylene).^202^

Other ongoing ML-based investigations of conventional polymer property prediction include identifying dopant-polymer conjugation combinations for synthesis, screening methods for polymer proton exchange membranes for fuel cells,^211^ and prediction methods for high temperature, high-bandgap polymer^212^ materials using the sequence of chemical building blocks in the monomer units in the dataset.^213^ These studies are representative of the status of many current polymer datasets available for use today.

Many currently available open-access databases are expansive, detailed, and state-of-the-art, but may be limited in terms of their inclusivity of biologically-derived polymers such as PHAs, complex copolymers or polymers combined with additives, or other biologically produced polymers, or may lack complex structural or physical feature sets for these polymers. Future work will ideally continue to expand on these existing efforts to include PHA biopolymers as well as conventional polymers in these highly detailed fingerprinting analyses, and also to include other property values for biopolymers that may be scarce such as a complete profile of optical activity, electrical, thermal, and mechanical properties where available.

Moving beyond the scope of conventional polymers, recent work has also been completed to expand into the biopolymer property prediction space using ML. These investigations have specifically investigated PHA polymers and other biopolymers using ML. In a study by Li and colleagues, the spectroscopic data of PHB production was used to develop a predictive model for understanding how much PHB may be produced biologically from corn stover.^214^ Investigations of PHA polymers have been completed using molecular fingerprints to predict glass transition temperature using deep neural networks,^215^ and expansions of this prediction of PHA glass transition temperature into both the PHA homopolymer and copolymer space.^216^ In addition, molecular dynamics simulations for predicting glass transition temperature have been generated in several studies^77,139,216^ and generating both glass transition and melting temperature predictions together.^150^ Examples of the polymers explored in these ML investigations include PHB and copolymers of PHB.

Other recent studies using ML for PHAs include predicting melting temperature and glass transition temperature in PHA homopolymers and copolymers, and have used many different ML techniques such as neural networks.^217^ For small datasets or those with many detailed features or noise in the data, other well-established algorithms such as random forest regression, support vector regression, gradient boosting, K-nearest neighbors, and polynomial and linear regression to generate the predictions have been shown to be very effective.^150,218^ Other polyester family members may be included in these studies to help with structural and physical characterization and performance improvements such as poly(butylene succinate), which may not necessarily be bio-based or biodegradable.^218^

The thermal properties of PHAs are inherently related to the polymer structure, and provide valuable insights into the structure–property relationship of the PSPP tetrahedron through ML. For instance, Bejagam and colleagues developed a multi-objective optimization workflow in addition to the property prediction model that also examines uncertainty quantification in specific formulations of PHAs, which may have less data available to contribute to the model.^150^ This study also examined wide composition ranges of PHB and copolymerized PHBV, as well as more complex mcl and lcl PHAs. Using results such as these, synthesis and characterization studies may be planned using these ML models to examine uncertainty in a more efficient workflow.

However, these implementations may be data-hungry and necessitate long periods of manual dataset cultivation, particularly for less common or custom variations of PHAs. One study by Gurnani and colleagues has begun these efforts to explore more efficient data-enabled prediction and screening methods using multi-task neural networks, such as using ML itself to learn the important features of a given dataset from a polymer repeat unit.^210^ This is in contrast to inputting the features manually, to minimize the time necessary to decode the features by hand.^210^

Despite the aforementioned activity in the area for ML-assisted functional polymer design for conventional polymers, applications of data-enabled methods in the field of biologically-derived polymers, including PHAs, have been rather scarce overall. A recent study by Kuenneth *et al.* represents a notable exception in this regard, which we detail here as it relates to the quantifiable nature of PSPP relationships.^219^ The goal of the study was to identify bioderived and biodegradable PHA-based alternatives for seven of the most widely used conventional petroleum-based consumer plastics.

**Fig. 4:**
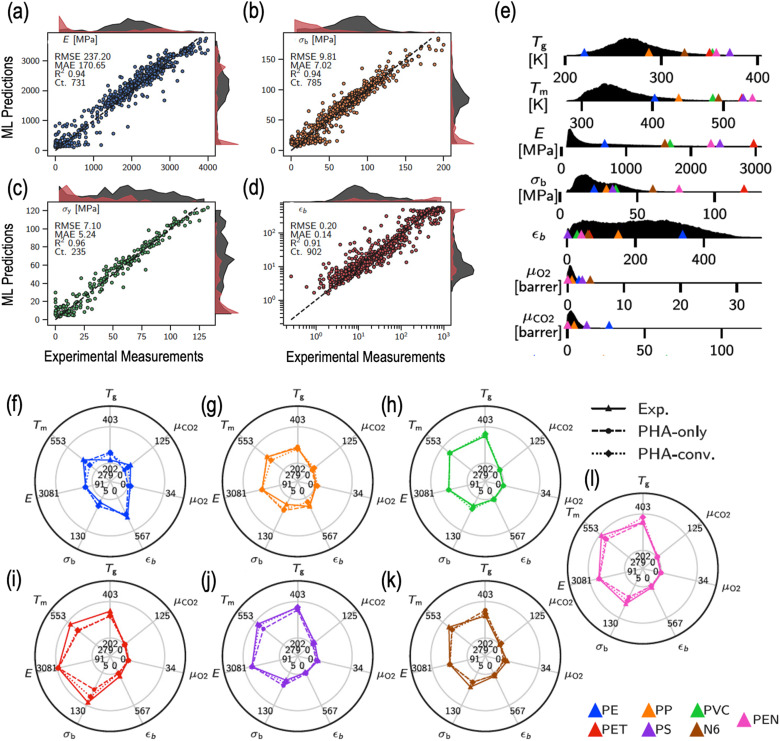
A representation of experimental and predicted properties of polymers using ML. (a–d) Parity plots of experimental measurements of a given property with respect to the ML predictions for that property, with root-mean squared error (RMSE), mean absolute error (MAE) and R-squared values listed on each panel (elastic modulus, stress at break, strain at break, and yield strength, respectively, clockwise from top left panel) (e) Density profiles for individual materials properties (including glass transition temperature, melting temperature, Young's or elastic modulus, stress at break, elongation or strain at break, and the gas transport and barrier properties of oxygen and carbon dioxide, respectively from top to bottom) computed over an entire dataset for prediction of bioplastic candidates. Missing x-axes beyond a certain cutoff indicate zero predicted property densities over those property ranges. Triangles represent the experimental property ranges of each of the seven commodity plastics listed in the bottom of the panel. (f–l) Property radar charts for each commodity plastic. Triangles with solid lines show the experimental properties. Circles with dashed lines and diamonds with dotted lines indicate predicted properties of the bio-replacements for the copolymer subgroups of PHA-only and PHA-conventional polymers, respectively. Reproduced from ref. 219 with permission from the Creative Commons Attribution 4.0 International License and Springer Nature publishers, copyright 2022.^219^

To accomplish this goal, the authors relied on a curated multi-property polymer dataset containing nearly 23 000 data points for 13 different properties (including thermal, mechanical and transport properties) to develop surrogate property prediction models.^219^ The dataset, spanning over a chemically diverse set of 15 344 homopolymers and 7512 copolymers, was employed to train and validate state-of-the-art multi-task deep neural networks to make accurate predictions of the target properties.^219^ As an example, Fig. 4a–d compare the prediction performance of the trained model with the corresponding experimental measurements for different mechanical properties, namely, Young's modulus (*E*), tensile strength at break (*σ*_b_), tensile strength at yield (*σ*_y_) and elongation at break (*ε*_b_).^219^

Subsequently, a truly large prediction dataset containing nearly 1.4 million PHA-based polymers was enumerated to screen and propose candidate replacement polymers exhibiting a property portfolio similar to any of the seven mainstream polymers.^210,219,220^ Property density profiles, for a selected set of selected thermal (glass transition temperature *T*_g_ and melting temperature *T*_m_), mechanical (*E*, *σ*_b_ and *ε*_b_) and transport (molecular permeabilities for O_2_ and CO_2_) properties, computed over the entire prediction set of bioplastic candidates are depicted in Fig. 4e, along with the experimentally-measured properties of the seven mainstream polymers shown by triangles.

Many conventional polymers fall at the high-end range of the spectrum of properties that PHAs may fall into. In some cases, only a small fraction of PHAs and PHA-based polymers exhibit a compatible range of properties to adequately replace conventional polymers, and sometimes this is achieved by pushing the polymer to its physical limit.^219,221^ This highlights the difficulty in identifying suitable replacements for the conventional polymers using the traditional trial-and-error-based approach, further emphasizing need for advanced polymer informatics approaches.^189^

With the property profiles available over the entire candidate set of 1.4 million compounds, a nearest neighbor search (within the target property space) was used to find several *closest* replacements for each of the seven conventional polymers.^219^ Moreover, in each case, the predictions were made for both PHA-only as well as PHA-conventional hybrid copolymers and their possible synthesis routes were discussed.^219^ As an example, Fig. 4f–l present qualitative graphical pairwise similarities between the property profiles of the identified top bio-replacements (PHA-PHA and PHA-conventional hybrid copolymers) and the conventional polymers in the form of radar plots to demonstrate that the identified replacements indeed exhibit similar sets of properties with respect to the experimental properties of the targeted polymers.^219^

While follow up experimental studies would be required to assess and validate these predictions, this study clearly demonstrates how informatics tools can help expedite design and development of bioplastics with improved thermomechanical and transport properties for better performance in specific applications. This study builds on other work within recent years in this rapidly accelerating field.^192,219^ Moreover, the approach can be readily extended to other polymer classes and augment conventional empirically-based design approaches by guiding the way to more targeted experiments, fewer experimental trials, or shorter times to market. This approach has potential for reducing the overall cost of PHAs. These recent activities in the field have demonstrated the ongoing enthusiasm and rapid acceleration of research scope in ML-assisted property prediction for polymers in general, and biopolymers such as PHAs in particular.

There are additional promising advances in the polymer informatics ecosystem in development. To help to establish foundations for building future ML-based property prediction and screening techniques, a recent report from Sahu and colleagues has provided an open-access Python toolkit using simplified molecular-input line-entry system (SMILES) strings of the polymer repeat unit as the primary input to tune the parameters of prediction and manage computational costs.^222^ Further research in this landscape has worked to harness existing ML techniques such as natural language processing to more efficiently build datasets such as PolyBERT as previously described, opening the door for future studies to continue to expand on these efforts.^200,223^ PolyBERT serves as one model for future studies that may take this foundation one step further to utilize autonomously-generated datasets of PHAs and other bio-derived polymers or naturally occurring polymers such as cellulose, starch, lignin, and others.

Providing a detailed framework for biomaterials for researchers unfamiliar with Python or other programming languages is a difficult task, but Meyer and colleagues have recently created a toolkit to familiarize researchers with basic ML techniques and how to implement them.^224^ The toolkit also provides an open-access tutorial Python coding script with the Google Colab platform. This approach is tremendously helpful for both data scientists and experimentalists alike to guide future studies.^224^ Not only is this a useful toolkit for advancing the field, but the design-build-test-learn paradigm outlined in this work also is a helpful way of understanding how ML can relate to biomaterials science and design. This represents one example of how the PSPP tetrahedron framework may be applied to implementing data science principles for furthering PHA study.

Another recent effort in the polymer informatics ecosystem by Chen and colleagues has further articulated current data acquisition and management strategies, polymer representation and prediction schemes, and given specific examples of successful implementations of property prediction and lab-based synthesis executions in the areas of polymer membranes, lithium ion batteries, and capacitors.^189^ To echo these exciting discussions, the use of experimental-data and simulation-generated data in a structured and findable, accessible, interoperable, reproducible (FAIR) manner is critical to enable more complex, realistic, and reliable ML-based screening tools which are repeatable by the community. A seamless integration of both computational and experimental workflows that integrate domain knowledge into experimental design will help to advance this area of ML development.^189,225^ Creation of user-friendly interfaces for experimentalists to report lab-acquired data will be helpful for streamlining the dataset development process in the future. Continued collaboration and discussion between experimentalists and data scientists will push the polymer informatics ecosystem forward.

## 4 Processing of PHAs

Processing in materials science is a broad term that encompasses the different ways a material may be changed after initial synthesis.^226^ For polymers, this term may describe a variety of approaches ranging from primary processing such as synthesis, extraction, and purification of the raw polymer to secondary processing such as extruding, casting, spinning, annealing, or blending the polymer into a suitable commercial product.^52,226^ In this section, we will consider both the differences in molecular weight and properties in: (1) PHAs of identical chemistry that undergo different physical processing steps, and (2) PHAs that have been blended to create composites with other materials. The latter will serve as the main focus of our present processing discussion due to this being the current most widespread method for tailoring the properties of PHAs after synthesis.^83,168^ Both topics also offer a huge range of potential for future research and exploration and are both similarly relevant in the commercial space, particularly for future research in which models of polymer processing suitability conditions may be developed with ML.

### 4.1 Processing of PHAs by chemical, thermal, and mechanical methods

The processing history of the material can directly impact the molecular weight and materials properties of two PHA polymers with the same structure and the same copolymer ratio.^80,169^ These processing steps may include methods commonly used with conventional polymers such as solvent casting or drop casting, extrusion, electrospinning, and hot pressing PHA granules.^119,141,144,227^ The extent of the difference between the final properties of the material will vary depending on the depth and variety of the processing history it is subjected to, as well as the conditions the material experienced during the collection of the material from the bioreactor or other synthesis source.^144,227^ Both of these processing history stages must be taken into account when examining the resulting properties.

Solvent or solution casting is one of the most widely used processing methods to create PHA films.^228–231^ In this approach, raw PHA granules are dissolved in an organic solvent and cast into a mold to prepare films of varying thicknesses.^230,232^ Chloroform is one of the most common solvents used for solution casting and glass molds are also one of the most typical mold types used in laboratory settings.^230,232^ Solvent casting can be used exclusively or in combination with another processing technique, such as electrospinning or hot pressing, the latter of which is common to prepare samples for mechanical testing.^230^ Other common fabrication and processing methods for developing PHA materials, particularly for packaging applications, include melt mixing, blending, or other compounding techniques, melt extrusion, compression molding, and electrospinning (particularly for blends with cellulose).^231^

Tanaka and colleagues recently illustrated the combined processing effects on the properties of PHAs. Solvent-cast films were heated with a hot press up to 30 °C above the melting temperature and crystallized isothermally to create narrow spherulites.^232^ This method was performed for both monomeric PHB, PHB-*co*-4HB, PHB-*co*-3HHx, and PHB-*co*-6HHx and compared with unheated control films. Molecular weight was reported to increase up to 279 kDa (PHB-*co*-3HHx) from 55 kDa (PHB) and 35 kDa (control PHB), and dispersity index was also shown to increase with the additional processing step adding the hot press.^232^ The film thickness and elastic modulus both increased significantly for hot pressed samples compared to those that were only solvent cast. The crystallization temperature was also shown to decrease by up to 40 °C above the starting PHB, along with a decreased degradation rate.^232^ In other solvent casting studies, drying in an atmosphere saturated with the solvent was used to achieve uniform mechanical properties and avoid internal stress formations.^144^

Building on the solvent casting technique, Kang and colleagues investigated oxygen plasma glow discharge after casting PHB films and the subsequent interaction with human fibroblasts.^233^ This process can be used to graft polyethylene oxide onto the film to introduce grafted acrylic acid groups and immobilize insulin for an attachment and proliferation study with human fibroblast cells for tissue culture. The control, unprocessed film and the oxygen plasma cleaned film were shown to have significant differences in cell attachment and proliferation due to the changes in hydrophobicity and roughness on the surface of the material. The prepared film was found to be similar to that of similarly prepared polyurethane and PLA substrates, presenting opportunities for bio-based alternatives with similar properties.^233^

In addition to plasma discharge, there has been work investigating how various other processing steps can improve the biological compatibility of bio-derived polyesters. Burkhardt and colleagues recently compared procedures for PHB, PLA, and polybutylene adipate terephthalate (PBAT) thermal annealing approximately 20 °C below the melting temperature.^234^ This was done to reorganize the amorphous regions of the semicrystalline polymers into more crystalline zones. The study examined the warping behavior of the polymers after steam sterilization at 134 °C, below the melting temperature of monomeric PHB, and demonstrated how mineral fillers such as calcium carbonate experienced reduced warping behavior during the process. In addition, the samples also exhibited accelerated degradation after steam sterilization.^234^

This example shows how the PSPP relationships may be taken into account for design. It represents the results of a combination of processing methods significantly changing the structure of the PHA. This change impacts the overall properties, and can lead to improved performance and biocompatibility of the PHA such as in extrusion-based manufacturing. By incorporating more than one aspect of the PSPP tetrahedron, experiments may be able to be more efficiently designed.^234^

### 4.2 Processing of PHAs by blending

To overcome the inherent property restrictions of naturally-occuring PHAs, one approach is to utilize copolymerization and blending of PHAs with other materials.^75,154,243^ For instance, a common method of PHA processing involves adjusting copolymer ratios of PHA-PHA copolymers during synthesis or in the initial pellet processing to adjust the thermal processing window. The overall melting temperature of the PHBV copolymer is lower than PHB monomer, and various copolymer ratios of HB and HV experiencing other processing histories including extrusion and compression (hot press) molding have been previously explored.^141,145^ In an example, Larsson and colleagues used a twin screw extrusion device to vary the melt temperature, rotation speed, and copolymer (PHB-*co*-P4HB) ratios and examined changes in blend morphology, thermal and glass transition temperatures, and viscoelastic properties.^141^ All samples experienced a decrease in the glass transition and melting temperatures, a decrease in the crystallinity, and an increase in the dynamic shear modulus (indicating a more elastic material) compared to the starting control material.^141^ A summary of the property changes that result from blends of PHB with other PHA monomers, other biomacromolecules, and various conventional polymers are provided in Table 2.

**Table 2:** A summary of PHB blend schema with other two other PHA monomers, several biopolymer materials, and various conventional polymers. A brief description of the subsequent properties that are affected in the blend compared to neat, homopolymeric PHB are also provided. This comparison provides a high-level trend regarding specific materials altering the physical properties of neat PHB *via* blending. Properties represented in the table include: melting temperature (*T*m), glass transition temperature (*T*g), elastic modulus (*E*), elongation (strain) at break (*ε*b), gas barrier properties for oxygen, and crystallinity (*X*c)

Blend Materials	Qualitative Summary of Property Changes	Ref.
PHB-4HB (with PBAT)	Decrease in *T*_m_, *X*_c_, increased *T*_g_, *ε*_b_, and *E*, and increased interfacial adhesion	75, 141 and 235
PHB-HHx (with PS)	Decrease in *ε*_b_, increase in *T*_m_, increase in opacity	75 and 236
PHB-HHx (with Nanocellulose)	Little to no decrease in *T*_m_ and *T*_g_, improved thermal stability, little to no increase in *E* and *X*_c_	75, 79 and 237
PHB-Lignin	Increase in *X*_c_, and *E*, decrease in *ε*_b_, *T*_m_, and *T*_g_	75, 76 and 179
PHB-Starch	Decrease in *E*, *ε*_b_, increase in *T*_m_ and *T*_g_	75, 83, 178, 237 and 238
PHB-PLA	Increase in *E*, *ε*_b_, *T*_m_, *T*_g_, and barrier properties (oxygen)	75, 82, 237 and 239
PHB-PVA	Decrease in *E* and decrease in overall mechanical durability	75 and 83
PHB-PET	Little to no increase in *E*, increase in *ε*_b_, increase in *T*_m_ and *T*_g_	75 and 83
PHB-PCL	Decreased *T*_m_, *T*_g_, and *E*, increased *ε*_b_	75, 86, 240 and 241
PHB-PS	Little to no increase in *E*, little to no decrease in *ε*_b_, increase in *T*_m_ and *T*_g_	83 and 242
PHB-HDPE	Little to no increase in *E*, increase in *ε*_b_, decrease in *T*_m_ and *T*_g_	83 and 155

These copolymer ratio adjustments performed by blending can dramatically change processing a PHA when utilizing existing conventional polymer processing equipment. This process ultimately dictates applications in which the material can be used.^151,235^ PHAs compatible with existing polymer processing equipment that can be used for other polymers as well are also less expensive. For example, monomeric or overly high ratios of hydroxyvalerate (HV) co-monomer (over about 40%) make the polymer overly soft, sticky, and unsuitable for processes such as injection-molding.^56,244,245^ Therefore, the copolymer ratio, both with other PHAs and with other materials, is typically seen as one of the most important processing factors for PHAs.^75,235^

In addition to PHA homopolymers and PHA–PHA organized block or random copolymers, there have been previous PHA processing studies that have altered the structure of existing PHA bio-based polymers with other types of biological materials.^76,177^ Some examples of such blended materials include PLA, starch, lignin, and cellulose as blends with commercial or lab-produced PHB to produce new, combined structures.^75,83^ Lugoloobi and colleagues utilized a blend of lignin and PHB developed using Pickering emulsion and formed films *via* thermal compression.^178^ The overall crystallinity of the film was increased as well as the elastic modulus and tensile strength while retaining a similar degradation rate to the base polymers, but making it more brittle than either in the process.^178^

Valentini and colleagues have explored the influence of blending with fibrillated nanocellulose on the degradation rate and thermal properties of PHBHHx.^79^ The authors produced the PHBHHx blend *via* ultrasonication and solution mixing with an increasing cellulose concentration ranging from 0.5 wt% to 3 wt%.^79^ Elastic modulus and tensile strength reached a maximum increase at the 0.5 wt% concentration, and decreased with increasing concentrations of cellulose, limiting the benefits of incorporating additional cellulose beyond that point. No influence on the thermal properties or degradation rate was observed with the addition of the nanocellulose.^79^ These studies highlight the need to better understand and quantify the interactions at the microscopic and mesoscopic level between PHAs and other bio-based polymers to develop a more consistent co-design relationship of how the additional biopolymer affects the structure of the blend.

Studies have explored changes in PHA structure by blending PHB with other materials, including synthetically made materials. For instance, biopolymers made by synthetic synthesis reactions such as polyvinyl alcohol, PEG, and PCL have been blended with PHAs. In addition, more traditional polymers such as PET, PS, and HDPE have also been blended with PHAs, and each has resulted in different biodegradation rates than the original neat homopolymers by themselves.^75,141,235^ These blending approaches aim to keep the original degradation profile of the material consistent without sacrificing the changes to the physical properties achieved through blending, such as mechanical strength and thermal stability. Enhancing the overall materials properties such as durability, elasticity, or expanding the thermal processing window are typically aims of blending. Retaining the degradation rate for specific applications is often also a consideration.^48,65,100,179^

For instance, Larsson and colleagues blended both homopolymeric PHB and block copolymer P(HB-*co*-4HB) (PHB4HB) with PBAT using melt extrusion.^141^ The melting temperature of the blend decreased with increasing PBAT composition, while glass transition temperature and shear storage modulus increased with increasing PBAT composition with a wide range of variability in sample results. However, there were distinct phase domains of homopolymer PHB visible in a continuous PBAT matrix. This result indicated immiscibility. Dicumyl peroxide, a compatibilizer, was added *in situ* during extrusion. The overall miscibility and blend properties of the polymer were improved by this addition.^141^

In another study, Tamiya and colleagues examined a blend of PHBHHx with PS that were grafted together to make a film that was shown to be heterogeneous, opaque, and brittle, showing similar immiscibility between PHAs and synthetic polymers.^236^ However, when modified using propargyl-terminated PHBHHx and azide-modified PS, the films exhibited improved homogeneity, transparency, and improved elastic modulus and tensile strength compared to the previous grafted blend or the PHBHHx alone.^236^ These processing studies indicate the need for an additional compatibilizer or Cu-catalyzed azide–alkyne cyclo-addition reactions. Both of these methods are very well understood processes, but these techniques may not always be environmentally sustainable or economical for scaling up the process in industrial and commercial settings.^180^

Single-layered PHB monomeric films have often been shown to be too brittle for mechanical analyses.^240^ To create a single-layered homopolymer film, PHB granules were blended with PCL by solvent casting. The as-cast film was then hot-pressed in a mold. Comparing untreated controls with the single-layered PCL film and heat treated multi-layered PHB-*co*-PCL polymers, the tensile strength and elastic modulus of the single layer PCL film was slightly higher, but the multi-layered copolymer film was shown to be more elastic with a higher yield at break, indicating shifts in the properties for PHA copolymer films depending on processing history.^240^ PHAs in general, and particularly homopolymeric PHB, are often blended with PCL or other materials in various forms to customize the thermal processing range, degradation rate, and mechanical strength of the blend by taking advantage of the different properties between the materials.^86^

Investigating potential processing techniques and optional blending strategies to achieve desired property ranges will be helpful for future work. A clear next step in developing the PSPP relationship between structure, properties, and processing for PHAs is to begin to establish these kinds of holistic quantitative relationships that connect each of these features. Some examples have already started to establish a framework for future investigations. These existing studies include explorations of film-forming processes and their effect on the resulting transport properties,^239^ the structural and property differences between wet-spun and melt-spun fibers,^246^ and the effects of thermal pre-processing steps on molecular weight changes.^243^

The physical way the PHA structure is altered during the processing stage from raw PHA granule to a usable product form is crucial to developing an understanding of how the overall materials design process can be streamlined for PHAs because of the direct impact on the material properties. For example, exploring the correlations between the number of carbons in the side chain and polyester backbone, the functional groups within each component of PHA, and how these factors influence the resulting properties of the material are all important considerations.

## 5 Performance of PHAs

The performance of a material is defined as its capability to meet a given set of requirements to function reliably in a desired role over a certain period.^51^ Performance metrics or criteria are often determined by its suite of properties and consequently how well suited the material is for the target application.^226^ In this regard the performance is usually discussed in the context of a particular application. In the context of PSPP relationships, many times the performance goal for PHAs will be to optimize the properties within the current standards of conventional plastic materials such that the PHA will serve as an effective alternative.

PHAs are currently most widely commercially produced for two broad categories of applications: biomedical products and food packaging or other consumer products.^135,183,247^ More recently, developments have also been made for PHA applications using additive manufacturing to improve their performance.^248^ This section aims to provide: (1) an overview of how PHAs are used in these three major fields, and some insights into other potential applications that extend beyond the scope of these three areas, (2) the performance metrics by which they are evaluated according to their properties and other PSPP relationships for each field, (3) and finally, present some opportunities for future research and additional areas of PHA applicability to continue to increase the performance potential of PHAs going forward in each field.

### 5.1 PHAs in biomedical applications

PHAs in biomedical applications are some of the widely researched areas of applications for PHAs. Biodegradability and non-toxicity are crucial properties that make PHAs ideal for use as biocompatible materials that break down in the body naturally, and relates to their physical structure and inherent properties. These properties have generated much excitement for their use in the biomedical field.^48,81,86,179^ Research on PHAs in various applications ranging from cardiovascular, nerve, bone tissue engineering constructs and drug delivery systems to artificial skin and beyond have been extensively explored. This research has been recently thoroughly reviewed in several recent reports.^86,249^

PHAs show other major biological advantages compared with traditional synthetic polymers in these spaces, with particular interest in their biocompatibility and low thrombogenicity.^81^ Furthermore, an additional benefit is their degradation mechanism since the local pH value during degradation of PHAs has been shown to remain unchanged.^118,250^ This makes them well tolerated by cells and the immune system compared to other clinically used polymers, such as PLA and PCL.^81,173^ Additional steps as described in the processing section of this review can further alter the properties of PHAs to tailor their performance to meet specific demands in these applications.

There are several representative performance metrics for PHAs as a biomaterial. PHB in particular has been previously evaluated for its ability to facilitate cell seeding, adhesion, proliferation, differentiation, and promote *de novo* tissue regeneration by Zinn and colleagues.^118^ In terms of providing physiological support, PHA copolymers have been investigated as a variety of tissue scaffolds (most notably for bone tissue engineering).^81,250^ PHBV-hydroxyapatite composites, for instance, have comparable physical and chemical similarities with human bones and serve as an excellent implant candidate for bone scaffolds and also recently, potential for artificial tendon composite materials.^63,251^

Not limited only to these applications, composite development of PHAs with bio-glass has also drawn recent research interest in the effort of perfecting the material for hard tissue engineering implants, as well as for PHA-bioceramic composites for bone drug delivery.^137,250^ In these examples, the major emphasis is often not on the thermal property processing ranges of PHAs (although performance related to local heating or ablation technology has been investigated), but rather on the trade-off between their degradation rate and mechanical durability as performance metrics.^172^

Soft tissue implants and regeneration platforms are also of particular interest for PHAs, demonstrating the diversity of potential PHA applications.^252,253^ Trakunjae and colleagues have recently demonstrated how the mechanical properties of PHBHHx can be tuned to eliminate surrounding healthy tissue modulus mismatch and promote skin tissue regeneration.^151^ A survey of recent work examining PHAs in tissue engineering by Pulingam and colleagues explored PHAs as wound dressing materials.^253^ The reported formulations of PHAs include incorporated gelatin, collagen, and keratin-impregnated P4HB electronspun nanofiber meshes and scaffolds. These impregnated meshes are used to promoted changes in both the hydrophobicity of the material as well as in its mechanical properties. Subsequently, these changes promoted adhesion and proliferation of murine fibroblast cells during *in vivo* studies. The results suggested up to a 35% increase in wound healing over 14 day studies compared to controls.^253^ In addition, the piezoelectric effect exhibited by PHAs has also been used for exploring nerve tissue regeneration using PHB scaffolds and induction of electrical properties by thermal or mechanical stress.^254,255^ Additional studies and uses of the piezoelectric effect in PHB will be discussed in section 5.4.

Other important performance considerations with regard to using PHAs as a biomedical material is in both their federal approval for use and encapsulation efficiency.^231^ The U.S. Food and Drug Administration provided approval for the use of PHB in absorbable sutures for surgical applications in 2007.^256^ This approval marked the progress of PHB meeting the general standards of accepted performance in medical and commercial use applications. Applications of PHB in various biomedical applications have been well-documented since then.^257–260^ In terms of their encapsulation efficiency, and therefore potential to be used as drug delivery agents, miscibility with various solvents is also an important performance feature specific to the biomedical use space.^250^

PHB was previously used to encapsulate a drug for controlled drug delivery, with the aim to adjust the material degradation rate over time to control the release kinetics of the compound.^81,250^ In studies to further the development of PHB nanoparticles to encapsulate the anticancer drug, docetaxel, Vardhan and colleagues as well as Luo and colleagues have exploited the hydrophobicity of PHB coupled with poly(lactide-*co*-glycolic) acid (PLGA).^261,262^ The encapsulation efficiency was shown to increase when a higher percentage of PHB was used, opening the path forward for future research to expand on these studies.^261^

There has been extensive research and reviews published in the area of PHAs for biomedical use, and it is a rapidly expanding field with a nascent commercial space.^253,263^ Future studies can benefit from input regarding a detailed quantitative PSPP relationship using the wealth of property data that already exists to optimize PHA materials selection, along with the incorporation of data science approaches such as ML datasets that can utilize or incorporate previously published literature reviewed for a particular application, such as absorbable sutures.^250^

### 5.2 PHAs as food (bulk) packaging and consumer plastics

The food packaging, food storage, and disposable products industries are currently the most extensively studied, economically viable, and fastest-growing fields for PHAs.^57,247^ Both PHB and PHBHHx were approved by the U.S. FDA for food contact in 2017.^274^ PHAs have been studied extensively and commercially used as everyday household disposable items such as bottles, food wrappers, diapers, pens, cutlery and utensils, and containers.^142,183,252,266,275^ Flexible PHA packaging materials, including bags, envelopes, pouches, and wraps made of easily yielding and deformable materials such as single-layer films, foils, or paper sheeting that can be filled and sealed are some other common examples.^86,136,266,275^ PHA polymers in food packaging are typically used to surround the contents completely, securing its contents from gases and vapors, moisture, and bacterial or other biological effects of the outside environment. These types of products are used as a barrier to keep dirt, germs, liquids or gases on one side of the film. This is an ideal example for utilizing the unique properties of biodegradable PHAs in consumer applications (summarized in Table 3).^57,238,266^

**Table 3:** A summary of four main groups of applications of PHAs. For each category of PHA application, several commonly-reported examples and the associated references are provided

Category		Common examples	Ref.
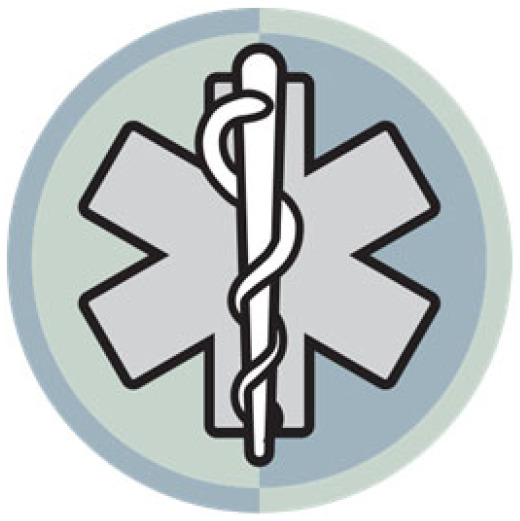	Biomedical applications	Drug delivery and encapsulation devices, bone tissue scaffolding, wound dressings	48, 63, 81, 86, 118, 137, 151, 172, 179, 249–253 and 258–264
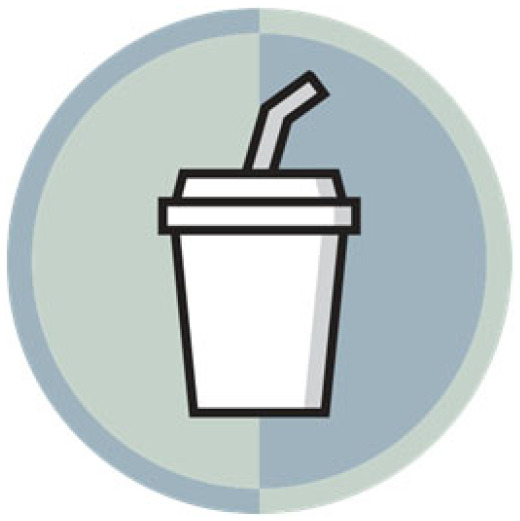	Food packaging and consumer goods	Single-use or reusable bags, envelopes, pouches, wraps, single- and multi-layer films, foils, sheets, boxes, disposable cups, plates, and cutlery	5, 70, 102, 135, 142, 183, 188, 237, 238, 265 and 266
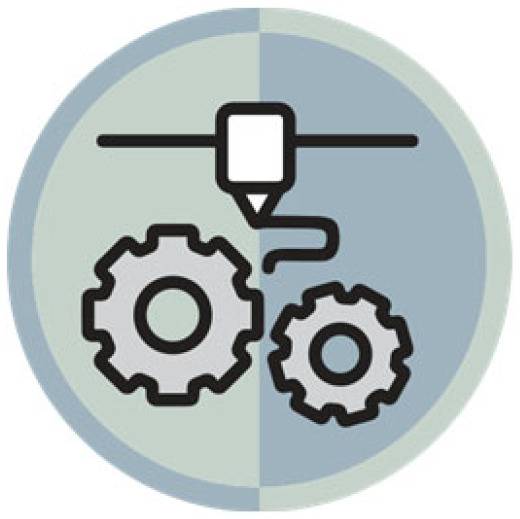	Additive manufactured PHAs	Fused deposition modeling, selective laser sintering, computer-aided wet spinning, stereolithography, blended materials for printing PHAs (PHA blends with poly(urethanes), alginate, lignin, beeswax, cellulose)	79, 82, 234, 241, 248 and 267–271
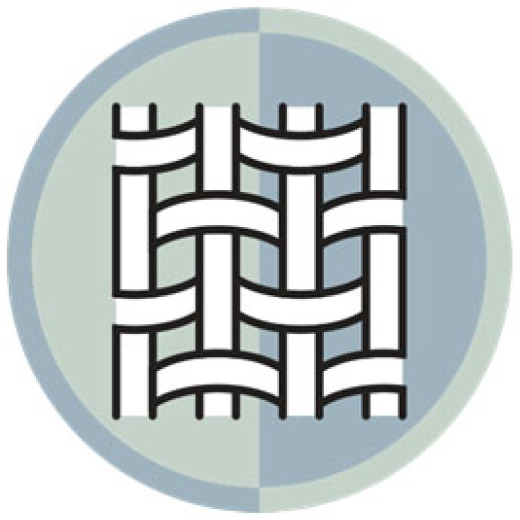	Other applications	Textiles and wearables, agricultural products (livestock feeds, mulch crop bags), automotive lubricants, jet and aerospace biofuels, cosmetic additives, electronic components, paint additives, biodegradable inks, fire retardants, disposable hygienic products	1, 28, 39, 48, 69, 83, 102, 239, 263, 272 and 273

There are several important properties and performance metrics considered for PHAs to be used in food packaging applications. These applications necessitate good barrier properties such as a low permeation rate, which is obtained with a combination of a thin film, low diffusivity, as well as low solubility and increased crystallinity, which relate to the structure of the material.^238,265^ Kumari and colleagues recently provided a detailed review regarding the current properties of sustainable packaging materials such as PHB and starch-based polymers.^237^ Diffusion coefficients generally decreased with increased HV copolymer content, that decreased the overall crystallinity.^237^ The physical barrier properties of PHA films have also been explored recently using the addition of inorganic laminar nanofillers such as nanoclays, or organic nanoparticles of starch and cellulose.^237,238^

These PHA composite films demonstrated decreased oxygen, carbon dioxide, and water vapor permeability rates as compared to LDPE, PP, PVC, and PS controls. These types of modifications to the structure of the PHA enhanced the barrier properties compared to the control conventional polymers.^237,238^ Note that the low permeability values for oxygen, carbon dioxide, and water vapor are generally of great interest when optimizing properties for food packaging, as discussed in section 3.4. In addition, another performance metric of interest is the degradation rate of the PHA, without the resulting toxic byproducts such as microplastics as is the case with conventional petroleum-based and other synthetic plastic, or the need for expensive industrial composting facilities as is the case for other commercially available bio-derived plastic alternatives.^57,136,275^

All property values for PHAs must fall within the performance metric ranges for conventional materials in order to be considered a suitable alternative. PHAs are required to at least meet or exceed the mechanical strength of the materials they would replace and be at least compatible with the same thermal processing ranges as the incumbant materials.^70,265^ This is often the case to assist with ensuring manufacturing and processing equipment capability for replacing the polymers with PHAs, and for practicality. In a report by Fabra and colleagues, crystallinity, thermal, mechanical, optical, anti-bacterial, and barrier properties were all examined for PEG-PHBV, PHB, and PHBV films over the course of three months for both single-layer and electrospun multi-layer food packaging prototypes.^265^ Multilayer films became stiffer, less deformable, and slightly more crystalline with higher barrier properties for oxygen transport than single-layer films across all samples, and storage time did not significantly alter these properties.^265^

Anti-bacterial and anti-viral studies of PHAs in various applications have been recently comprehensively surveyed by Ladhari and colleagues.^231^ These studies have explored the potential for agents and processing procedures that may be incorporated into PHA design to enhance performance. Examples of these steps for anti-bacterial treatment include plasma cleaning or nanofibers integrated with anti-bacterial agents. Protonated amino groups, nanoparticles, and other substances may serve as these anti-bacterial agents.^231^ This indicates that the holistic effects and PSPP relationships for each set of properties and the processing steps leading to the final commercial product are important to take into consideration when designing food packaging materials using PHAs.

The inherent PSPP relationships of PHAs have provided several advantages over other competing bio-derived materials. However, current design challenges such as economic feasibility and commercial scaling for food packaging in particular still hinder widespread use of PHAs as alternatives to conventional plastic materials. PHA performance as it relates to the structure, properties, and processing history of the product may be more thoroughly examined in future studies using ML and polymer informatics to approach co-design principles in a more quantitative, comprehensive way. As discussed in detail earlier in section 3.5, there have been recent efforts towards establishing a method for multi-property ML predictions specifically targeted for screening PHAs to compare with other conventional and other bio-based plastics for common commercial applications.^219^

To design PHAs and other bio-derived materials that can reliably perform in the commodity plastics market, the PSPP relationships must be established in these ways. This type of research approach, incorporating polymer data informatics and ML together with real-world experimental validation data, represents a step forward in the future of streamlining the design process for more commercially viable PHA design and development.

### 5.3 Additive manufacturing of PHAs

One of the most exciting areas of ongoing research in PHA materials design is in additive manufacturing.^248^ The existing additive manufacturing techniques that have been examined in detail for PHA manufacturing as of 2023 include fused deposition modeling (FDM), selective laser sintering, computer-aided wet-spinning, and stereolithography, that are described extensively in another review with specific PHA materials and PHA blends highlighted.^248^ In brief, these approaches typically use monomeric PHB, PHBV copolymers, and PHB-lignin blends for stereolithography and FDM, and PHBHHx for computer-aided wet-spinning.^248,267^

While PHAs can be currently processed by conventional polymer processing methods such as extrusion (with some existing major limitations), additive manufacturing offers some unique potential solutions to the existing challenges and narrow thermal processing windows for PHAs. For instance, one recent study employed fused filament fabrication with different polyester blends, including PHB, to improve the thermal characteristics of the material after printing, annealing, and sterilization, demonstrating significantly improved mechanical features and durability in the end products.^234^

One of the most widely-regarded advantages of additive manufacturing techniques that cannot be easily achieved by other methods is that it provides the on-demand fabrication of tailor-made products.^268^ Particularly in the biomedical space, additive manufacturing offers means to develop customized products for tissue regeneration, such as in cardiac tissue, cartilage and vascular repair, and wound dressings, in addition to promising therapeutics for hard tissue regeneration.^268^ Developing PHAs with additive manufacturing is advantageous due to this highly customizable nature of the polymer. There is a wide range of high-resolution geometries, rapid prototyping for patient-specific needs, and custom property value ranges that might otherwise necessitate resource-intensive handmade molds.^82^

Although there are exciting advantages for using PHAs with additive manufacturing, there are some environmental challenges that present opportunities for future research. To be a fully circular loop for sustainability, the additive manufacturing process requires the use of PHA and other material filaments, inks, and resins, that must utilize the properties of PHAs in order to be fully biodegradable.^82^ However, the energy, computational cost, and other support materials required by these processes offer other areas for future research to expand on the sustainability of using additive manufacturing for PHAs.^234,267^ Many of the other limitations for additive manufactured PHAs are based on their performance, that is currently constrained by the inherent structure and physical properties of the PHA or PHA blend. For instance, PHB and its copolymers are often limited by their compressive strength, thermal processing ranges, solubility, and cell adhesion, cell differentiation and specialization, and cell proliferation rates.^267,268^

However, there have been novel approaches to overcoming these limitations while maintaining the benefits of additive manufacturing that have utilized awareness of PSPP relationships. For instance, Lemos de Morais and colleagues have described methods in additive manufacturing for biopolymers. Specifically, a broad scope of research covering the filaments for raw materials for use in 3D printing, inks, and resins based on biomaterials or other renewable resources was reported.^267^ The discussion of property selection for the starting materials such as inks and filaments combined with the processing variables was of particular interest from a PSPP relationship perspective.

Using methods described in various studies throughout their report, overall performance of additive manufactured PHAs were improved compared to neat PHB in solvent-cast films, particularly with regard to uniformity of the final material.^267^ For example, melt blending of PHBV with 20 wt% calcium sulfate hemihydrate followed by additive manufacturing with an FDM system significantly improved the rigidness and hydrophilicity of the PHBV scaffolds. These methods provide an approach to overcoming property limitation challenges in porous scaffolds for hard tissue engineering.^267^ The ability to tailor the geometry of the final products, tune the mechanical properties, and improve hydrophilicity for cell compatibility have all been reported as advantages of additive manufacturing for PHA biopolymers and blends.^82^ Several cases of these blends, such as with alginate, lignin, beeswax, cellulose, and poly(urethane acrylate), have demonstrated improved performance in specific biomedical applications.^82,269^

In addition to blends, composites of PHA materials with other materials have demonstrated potential in additive manufacturing. Novel PHBHHx-fibrillated nanocellulose biodegradable composites for additive manufacturing have been produced and characterized by Valentini and colleagues.^79^ Composite filaments having a nanofiller concentration of 0.5 wt%, 1 wt% and 3 wt% were then extruded, characterized and used in fused deposition modeling.^79^ The presence of fibrillated nanocellulose did not affect the thermal degradation behavior of the materials, nor were the glass transition and the melting temperatures influenced.^79^ This study marked a promising approach to overcoming the current limitations of PHA processing by utilizing new polymerization techniques in combination with other biopolymers (such as nanocellulose used here).^79^

Additive manufacturing of PHAs is an emerging field of study that is rapidly expanding. Within the past five years, many studies have established specific methods such as fused deposition modeling to assist in tailoring PHAs and PHAs blended with other materials to achieve specific mechanical properties and improve their overall performance, particularly in biomedical applications. However, these studies have also highlighted the need for continued investigation to further connecting the PSPP relationships and expand the field by continuing to relate the structure of blended PHAs to their properties with these novel processing techniques.

### 5.4 Other applications of PHAs

The scope of PHAs for consumer applications is not limited to the areas of food packaging, biomedical applications, or additive manufacturing alone. In fact, the use of PHAs continues to expand into a wide variety of commercial sectors. PHAs have been studied and utilized in various industrial applications such as textiles, agricultural products (*e.g.*, livestock feeds and mulch bags for crop production), automotive lubricants, biofuels for aerospace, cosmetic additives, electronics components, paint additives, biodegradable ink for printing disposable packaging, and adhesives.^28,272,273^ Studies have also utilized the anti-adhesion and anti-fouling nature of hydrophilic PHBs to: (1) suppress biofilm development in aquaculture facilities to decrease bacterial contamination, (2) improve the sweat-wicking, texture, and degradability of certain textiles and clothing products, and (3) develop wearable biomolecule sensors by modifying the functional groups of the PHA side chain.^28,239,263^ PHAs with a low viscosity have been used for injection molding to produce biodegradable diapers and other disposable hygienic products.^28^

PHAs are a unique biopolymer material in that they exhibit a piezoelectric effect not often reported in other polymers such as PLA or PCL.^255,276–278^ The piezoelectric effect in PHB has been well-documented for nearly four decades.^279^ PHAs have been investigated for use in components of electronic devices such as headphones, microphones, and atomizers.^28,48,264^ Due to their biocompatibility, PHAs have also been investigated for potential applications in flexible or wearable electronics,^255,277,278,280^ recently for their potential for nerve tissue regeneration and nervous tissue engineering as a piezoelectric soft material,^254,255,276^ and for their bacteriostatic effects.^281^ These studies build the foundation of this unique effect in PHAs which may further lead to other interesting applications such as soft piezoelectric devices in measurement tools or characterization equipment with future study.

Recent work has also investigated PHAs as a potential fire retardant by aiming to improve the thermal properties of PHBV copolymerized with poly(butylene adipate-*co*-terephthalate). Combined with metal oxide nanofillers as a stabilizer, these copolymers have been shown to display both good flame retardancy and mechanical properties.^28^ This is another exciting and unique feature that distinguishes PHAs from other types of commercial biopolymers. Finally, other recent studies have included exploring PHAs for use in biocomposites and as nanocomposites or nanoparticles with other inorganic and organic materials with the goal of improving the overall degradation and biocompatibility of the desired applications.^28^ There is much potential for future expansion into other applications not listed here. Future work will certainly utilize the relationships between the structure, properties, processing, and performance of PHAs for a more comprehensive understanding of biopolymer design and to explore these yet undiscovered applications.

## 6 Discussion and future outlook

In traditional implementations of the materials science tetrahedron, the PSPP relationships have been essential for not only providing a framework for an improved understanding of the fundamental concepts, but also for establishing best practices towards the rational design and optimization of novel and existing materials.^51,52^ In this review, we have leveraged this classic framework towards discussing the potential of future research with PHAs. We have started the discussion of bringing together cutting-edge data science techniques with previous decades’ worth of thorough PHA research in an interconnected and coherent way as previous studies have done with the digital version of the PSPP tetrahedron.^51^

PHAs present an excellent opportunity for future sustainable materials design and exhibit diverse chemistries with tunable properties that are of interest in a broad range of potential applications. It is imperative that the community continues to investigate the entire holistic co-design challenge in order to address the current issues pertaining to economic viability and property trade-offs/limitations with future research.^238^ The COVID-19 worldwide crisis has also highlighted in recent years the need for more systematic approaches and collaboration from industry, governments, and consumers to address the issue of plastic production, recovery, and degradation.^7^

However, the use of sustainable raw material and resources alone does not make a material sustainable. Holistic bio-derived polymer sustainability involves a variety of factors including economic feasibility, scalability with existing or new equipment for synthesis and processing, technical feasibility, and overcoming biological limitations. The economic demand and costs of each of these challenges must also be addressed.

Therefore, future research will also need to incorporate an analysis of these challenges and address current bottlenecks to identify innovative solutions to these problems throughout the entire life cycle of the material, not just for PHAs, but for other classes of future bio-derived materials as well. PHAs are still currently too expensive to be economically sustainable for widespread use in multiple applications. We suggest that the next steps for PHA materials development must incorporate a holistic approach to design by taking into consideration the PSPP relationships outlined in this review in order to reduce these costs.

The concept of “co-design” principles must be paramount going forward. For example, future work investigating the cost-effectiveness of the PHA production process must take into account the genetic and biological compatibility of the PHA producing organism strains, carbon sources, and the fermentation and bioreactor processes. These future studies should also take into account the utilization of waste feedstock, raw material processing, and resource recovery features all within the same experimental (or data-enabled) framework to design PHAs that possess the desired property portfolio for a target application.^28^

For instance, previously examined biosynthesis factors and production potential of PHAs using different carbon sources, microorganisms, and nutrient limitations as experimental factors have been well-documented on their own individually and in relation to each other from a biosynthetic standpoint.^20,86,100,125^ However, as with the structural and morphological aspects of PHAs and their relationship to PHA properties, a quantifiable, comprehensive relationship that considers each of the three major PHA-structure biosynthesis factors (microbe type, carbon source, and nutrient limitations) and the resulting PHAs that each combination produces has not yet been fully established.

Clarifying these biosynthesis factors which affect the structure of PHAs has the potential to enhance the overall design process of PHAs by establishing a PSPP relationship between how the material is biologically or synthetically prepared, its resulting structure, and the subsequent properties. Future studies would benefit from establishing such a relationship. Continued exploration will ideally also consider cost-effective biosynthesis techniques to access and control these specific structures.

With regard to the use of data-enabled design and ML, this is one area that we place an emphasis on understanding the experimental implications and physical meaning of results. Ideally, ML may be implemented in future work to streamline and optimize laboratory processes and synthesis schemes. We recommend a goal of tailoring the desired properties of PHAs specifically to meet desired property ranges for optimized performance in desired applications using these existing design strategies in polymer informatics with an awareness of PSPP relationships.^189^

Future research may also seek to break down the PSPP co-design principle into even smaller pieces, to still examine ways to expedite PHA research and development in more simple ML models. Such future explorations may include: (1) a surrogate model-based prediction using ML, and generated by polymer- and bio-informatics models, to develop a predictive and quantitative understanding of the PSPP relationships, (2) an experimental design approach that leverages active learning and informatics-based approaches to more efficiently investigate and validate theoretical predictions of the thermal, mechanical and other relevant properties, (3) the design of possible models and predictive analysis of optimized processing conditions for PHAs and other biopolymers to ensure their compatibility with existing industrial polymer processing equipment, and (4) further experimentation and ML model combinations incorporating novel or enhanced microorganism strains to improve the PHA productivity and yield *via* genetic engineering.^28,239^

Increased focus on environmentally-friendly production and processing processes, from synthesis and fermentation to more efficient and reliable downstream recovery strategies are still needed, and are currently open areas for future study and exploration.^273^ The thermal properties, mechanical durability, and barrier properties of PHAs must be investigated in tandem, rather than in isolation, as per the co-design principles of the materials tetrahedron to develop a more quantitative understanding of PSPP mappings. In this way, researchers may be able to address that processing conditions are ultimately suitable to achieve the target performance. In addition, the economic sustainability of each of these interconnected aspects of the materials design process must also be considered.^5,238^

Despite several outstanding challenges that currently exist as road blocks towards commercialization and widespread use of PHAs, the future of this class of biopolymers remains bright. There is tremendous potential for PHAs to become a mainstream alternative to conventional plastics in a wide range of applications as both an environmentally and economically friendly material. The community has an exciting opportunity to utilize state-of-the art experimental methods and novel polymer informatics approaches to create a new framework for understanding bio-derived polymers with a PSPP-focused perspective to create timely and impactful solutions to global concerns for everyday life.

## Author contributions

J.L. prepared and organized the manuscript and provided references. B.L.M. provided references, input, and structure. G.P. provided references, input, structure, and assisted with manuscript and figure development. All authors have worked together to conceptualize, read, and edit the review, and all have agreed to the published version of the manuscript.

## Data availability

All data and research presented in this manuscript represent the work of a review paper with full citations to all original research presented and clearly indicated. All data presented in this research is open access and available for access upon redirecting to the appropriate reference and resource.

## Conflicts of interest

There are no conflicts to declare by the authors.
